# Dilp8 requires the neuronal relaxin receptor Lgr3 to couple growth to developmental timing

**DOI:** 10.1038/ncomms9732

**Published:** 2015-10-29

**Authors:** Andres Garelli, Fabiana Heredia, Andreia P. Casimiro, Andre Macedo, Catarina Nunes, Marcia Garcez, Angela R. Mantas Dias, Yanel A. Volonte, Thomas Uhlmann, Esther Caparros, Takashi Koyama, Alisson M. Gontijo

**Affiliations:** 1 Integrative Biomedicine Laboratory, CEDOC—Chronic Diseases Research Center, NOVA Medical School | Faculdade de Ciencias Medicas, NOVA University of Lisbon, Campus do IGC, Rua da Quinta Grande, 6, Oeiras 2780-156, Portugal; 2 Instituto de Investigaciones Bioquímicas de Bahía Blanca (INIBIBB), CONICET and Universidad Nacional del Sur, Camino La Carrindanga km7, Bahía Blanca B8000 FWB, Argentina; 3 Dualsystems Biotech AG, Grabenstrasse 11a, Schlieren CH-8952, Switzerland; 4 Departamento de Medicina Clínica, Facultad de Medicina, Universidad Miguel Hernández, Ctra. Alicante-Valencia, km 87, San Juan, Alicante 03550, Spain; 5 Development, Evolution and the Environment Laboratory, Instituto Gulbenkian de Ciência, Rua da Quinta Grande, 6, Oeiras 2780-156, Portugal

## Abstract

How different organs in the body sense growth perturbations in distant tissues to coordinate their size during development is poorly understood. Here we mutate an invertebrate orphan relaxin receptor gene, the *Drosophila Leucine-rich repeat-containing G protein-coupled receptor 3* (*Lgr3*), and find body asymmetries similar to those found in *insulin-like peptide 8* (*dilp8*) mutants, which fail to coordinate growth with developmental timing. Indeed, mutation or RNA intereference (RNAi) against *Lgr3* suppresses the delay in pupariation induced by imaginal disc growth perturbation or ectopic Dilp8 expression. By tagging endogenous Lgr3 and performing cell type-specific RNAi, we map this Lgr3 activity to a new subset of CNS neurons, four of which are a pair of bilateral *pars intercerebralis* Lgr3-positive (PIL) neurons that respond specifically to ectopic Dilp8 by increasing cAMP-dependent signalling. Our work sheds new light on the function and evolution of relaxin receptors and reveals a novel neuroendocrine circuit responsive to growth aberrations.

## Abstract (web summary)

The orphan ligand Dilp8 has been shown to coordinate growth and developmental timing in *Drosophila*. Here, using Gal4 drivers and CRISPR/Cas9 approaches, Garelli *et al.* identify a role for relaxin-like receptor Lgr3 in regulating the Dilp8 developmental delay pathway.

## Graphical abstract

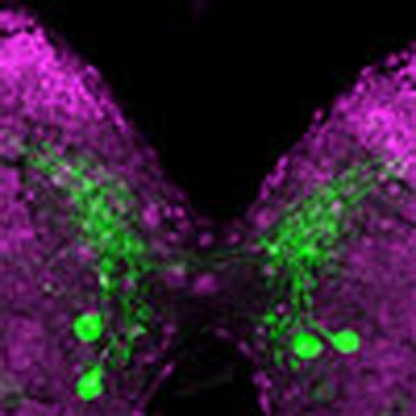

How different organs in the body sense growth perturbations in distant tissues to coordinate their size and differentiation status during development is poorly understood^1^,^2^. We have previously discovered a hormone in *Drosophila*, the insulin/relaxin-like peptide (Dilp8), which ensures organ and body size coordination^3^. In developing larvae, Dilp8 is produced and secreted from abnormally growing imaginal discs. Its activity transiently delays the onset of metamorphosis by inhibiting the biosynthesis of the major insect molting hormone ecdysone by the prothoracic gland, a part of a compound endocrine structure called the ring gland^3^,^4^ (Fig. 1a). Loss of *dilp8* uncouples the endocrine communication between imaginal discs and the prothoracic gland, making *dilp8* mutants susceptible to uncoordinated disc growth when intrinsic errors of development or noxious environmental stimuli affect the growth status of one or more discs. This results in an increase in random deviations from bilateral symmetry (fluctuating asymmetry (FA)), measurable at the population level^3^. These findings have placed Dilp8 as a central player in the interorgan communication system that mediates plasticity to promote developmental stability in *Drosophila*^3^,^4^,^5^,^6^,^7^. However, which molecule(s) and tissue(s) sense and/or transmit this abnormal growth signal to the prothoracic gland are unknown.

Type C1 Leucine-rich repeat-containing G protein-coupled receptors (Lgrs) are a conserved protein family in metazoans that act as receptors for insulin-like peptides of the relaxin subfamily in vertebrates, where they play diverse roles in tissue homeostasis and remodelling, behaviour and reproduction^8^. In invertebrates, however, they are considered orphan receptors and their biological function is a mystery because invertebrates are thought to lack *bona fide* relaxin peptide homologues^8^,^9^,^10^. The *Drosophila* genome encodes two orphan receptors, Lgr3 and Lgr4, with clear homologies to vertebrate relaxin receptors (∼45 and ∼40% sequence similarity to human RXFP1/2, respectively^9^,^10^). Here to gain insight into the function of invertebrate relaxin receptors, we mutate *Lgr3* and study its phenotype. We find that *Lgr3* plays a critical role in the Dilp8-dependent developmental stability pathway by acting in a new subpopulation of central nervous system (CNS) neurons.

## Results

### Lgr3 couples growth to developmental timing

**Figure 1:**
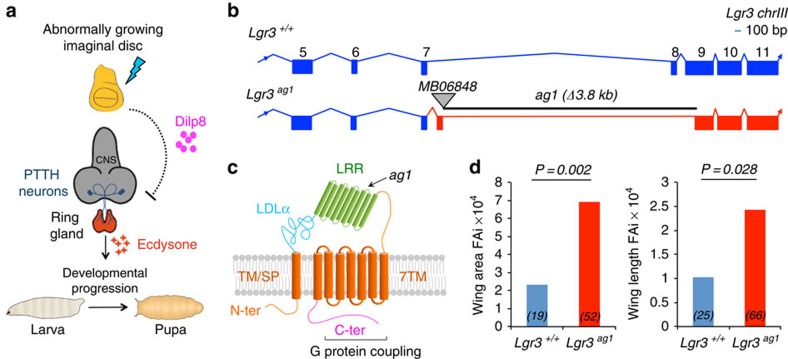
**Mutation in the** ***Drosophila*** **relaxin receptor Lgr3 leads to increased fluctuating asymmetry.** (**a**) A neuroendocrine pathway coupling growth and developmental timing^3^,^4^. Scheme adapted from Halme *et al.*^12^ (**b**) Remobilization of Minos element *MB06848* positioned between exons 7 and 8 of the *Lgr3* locus on chromosome III generated a 3.8-kb deletion, named *Lgr3*^*ag1*^. Reverse transcriptase PCR assays followed by Sanger sequencing determined that the *Lgr3*^*ag1*^ deletion leads to usage of an aberrant splicing acceptor within intron 7 and readthrough directly into exon 9 (Supplementary Fig. 2a–d). (**c**) Scheme of the predicted protein structure of the wild-type Lgr3 protein and the truncated Lgr3^*ag1*^ protein based on vertebrate relaxin receptor structure data^8^. Major domains are depicted: low-density lipoprotein receptor domain class A (LDLa), leucine-rich repeat (LRR) and seven transmembrane (7TM). The first TM/signal peptide (SP) domain is not predicted to be cleaved. The approximate region where *ag1* mutation truncates the Lgr3 protein is depicted. (**d**) Bar graphs of the FAi^3^ of the area (left panel) and length (right panel) of the wing pairs of the genotypes indicated. Numbers (*N*) of the wing pairs scored. F-test *P* values are shown. Wing area: degrees of freedom (df)_(ag1)_=51, df_(+/+)_=20, *F*=3.23; Wing length: df_(ag1)_=65, df_(+/+)_=28, *F*=2.08.

**Figure 2:**
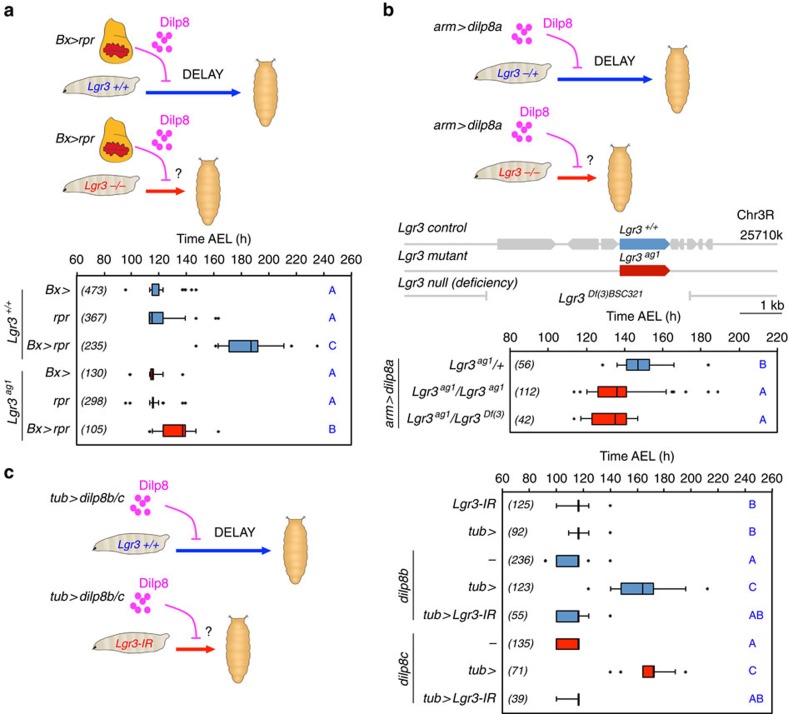
Lgr3 couples imaginal disc growth to developmental timing by acting in the Dilp8 pathway. (**a**) Mutation of *Lgr3* abrogates interorgan communication between regenerating damaged discs (*Bx>rpr*) and the neuroendocrine centers coordinating the onset of metamorphosis. (**b**) *Lgr3* acts in the Dilp8 pathway. Placing *Lgr3*^*ag1*^ over a deficiency uncovering the *Lgr3* locus suppresses the delay caused by ectopic *dilp8* expression (*arm>dilp8a*). (**c**) RNAi against *Lgr3* (*Lgr3-IR*) suppresses the delay caused by ectopic expression of either *dilp8b* or *dilp8c* transgenes^3^ under the control of the ubiquitous *tub>* driver. (**a**–**c**) Box plots (see Methods) showing pupariation time (Time after egg laying (AEL) in h) of (*N*) larvae obtained from six, two and six egg layings for panels (**a**–**c**) respectively. Whiskers are 5 and 95% percentiles. Dots, outliers. *P<*0.0001, Kruskal–Wallis test for all panels. Genotypes sharing the same letter (blue) are not statistically different at *α*=0.01, Conover *post hoc* test. Degrees of freedom, *H* and *C* values for the Kruskal–Wallis tests are df=5, *H*=666.45, *C*=1.28; df=2, *H*=54.16, *C*=0.99 and df=7, *H*=468.05, *C*=0.83 for panels (**a**–**c**), respectively.

To generate a mutant for *Lgr3*, we remobilized an MB Minos element^11^ (Fig. 1b) and obtained three precise excisions (*Lgr3*^*+/+*^), which served as genetic background controls, and one imprecise excision allele, *Lgr3*^*ag1*^, which consists of a 3.8-kb deletion that completely removes exon 8 and partially removes exon 9 (Fig. 1b and Supplementary Fig. 1a–d). *Lgr3*^*ag1*^ produces a transcript with a premature termination codon (PTC) that truncates the Lgr3 protein one amino acid after D_326_ (Fig. 1b,c and Supplementary Fig. 2a–d). We conclude that *Lgr3*^*ag1*^ encodes a severely compromised protein that is unlikely to bind ligand or signal due to a truncated ligand-binding leucine-rich repeat domain^8^, and absence of the seven transmembrane (7TM) domains and G protein coupling carboxy terminus (Fig. 1c). We noticed increased FA in *Lgr3*^*ag1*^ adult wings. FA indexes (FAi) are increased by an order of ∼3 when compared with their *Lgr3*^*+/+*^ controls (*P=*0.0028, F-test for wing area FAi (Fig. 1d)). This phenotype is indicative of uncoordinated imaginal disc growth during the larval stage, and is reminiscent of the increased FA phenotype of animals lacking Dilp8 (ref. 3). We thus hypothesized that Lgr3 and Dilp8 act on the same pathway to promote developmental stability by coupling imaginal disc growth to developmental timing. To test this hypothesis, we asked the question whether *Lgr3* mutants show a similar defect as *dilp8* mutants in the ability to delay the onset of metamorphosis in response to induced abnormal tissue growth^3^,^4^. We did this by inducing tissue damage in an *Lgr3*^*ag1*^ background and carried out pupariation timing assays. Tissue damage was induced by placing the proapoptotic gene *reaper* (*rpr*) under control of the wing-pouch *Beadex-Gal4* (*Bx>*) driver^3^,^12^, a combination that caused a 72-h (median) delay in pupariation in control *Lgr3*^*+/+*^ animals (Fig. 2a). This delay was reduced by 50 h in *Lgr3*^*ag1*^ mutants (∼80%, *P<*0.01, *post hoc* test after Conover for Kruskal–Wallis test (hereafter, Conover *post hoc* test); Fig. 2a). This demonstrates that Lgr3, like Dilp8, mediates the communication between abnormally growing imaginal discs and the prothoracic gland, strongly indicating that both proteins act on the same pathway.

### Lgr3 acts in the Dilp8 pathway

If Dilp8 and Lgr3 are in the same pathway, then *Lgr3* should be necessary for the developmental delay produced by ectopic Dilp8 expression in the absence of tissue growth abnormalities^3^,^4^. Indeed, the developmental delay induced by ubiquitously driving a *UASP-dilp8::3xFLAG* (*UAS-dilp8a*) transgene under the control of *armadillo-Gal4 (arm>)* was suppressed in larvae homozygous for *Lgr3*^*ag1*^ or trans-heterozygous for *Lgr3*^*ag1*^ and a deficiency that completely uncovers the *Lgr3* locus (*Lgr3*^*Df(3)BSC321*^) (*P<*0.01, Conover *post hoc* test; Fig. 2b). These results show that animals lacking Lgr3 are insensitive to ectopically produced Dilp8. Furthermore, as the suppression phenotype of *ag1* is indistinguishable from the *Df* allele, the results bring further evidence that *ag1* is a strong loss-of-function allele. To test whether Lgr3 activity is required for the Dilp8*-*dependent delay in an independent experimental setting, we induced a developmental delay using two previously reported *dilp8* transgenes (*UAST-dilp8::3 × FLAG* (*UAS-dilp8b and c*))^3^ under the control of another ubiquitous driver, *tubulin-Gal4* (*tub>*), and reduced *Lgr3* activity by concomitant RNA intereference (RNAi) knockdown using a short hairpin (TRiP VALIUM22 (*Lgr3-IR-V22*)) line^13^, which reduces the steady-state *Lgr3* mRNA levels by ∼85% (*P=*0.043, Student's *t*-test) (Supplementary Fig. 3a). Coherent with the mutant analyses, RNAi against *Lgr3* completely suppressed the *dilp8-*dependent delay (Fig. 2c). A second RNAi line producing a long hairpin against *Lgr3* (TRiP VALIUM10 (*Lgr3-IR-V10*))^14^ also rescued the *dilp8-*dependent delay, albeit to a lesser extent than *Lgr3-IR-V22* (Supplementary Fig. 3b). This partial rescue was proportional to a weaker reduction in *Lgr3* mRNA levels (∼50%) than *Lgr3-IR-V22* (Supplementary Fig. 3a), suggesting that the Dilp8 delay is sensitive to *Lgr3* dosage. The *Lgr3-IR-V22* RNAi transgene was hereafter used in further experiments, as it phenocopies the *Lgr3* mutant phenotype best and gives the strongest mRNA reduction. Together with the *Lgr3* mutation analyses, the *Lgr3* RNAi experiments strongly place Lgr3 in the Dilp8-dependent developmental delay pathway.

### Lgr3 is expressed in the CNS

**Figure 3:**
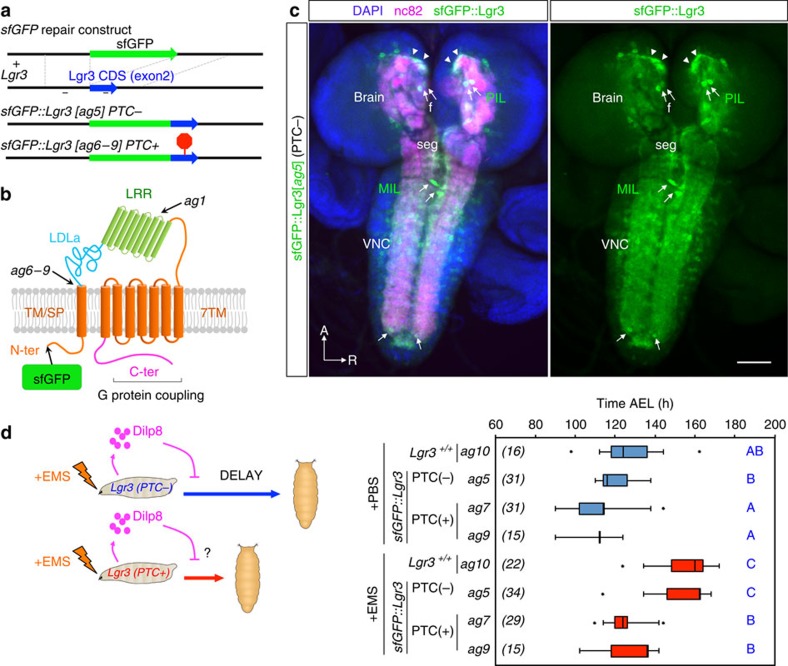
Lgr3 is expressed in a subpopulation of CNS neurons. (**a**) CRISPR/Cas9-mediated sfGFP knock-in strategy used to generate the Lgr3 protein reporter allele *ag5* (named as *sfGFP::Lgr3*), which does not contain indels in the exon 2 region, and alleles *ag6-9*, which contain PTC+ indels (Supplementary Fig. 4). Two thin black lines indicate the sites of the CRISPR gRNAs used. (**b**) Lgr3 protein scheme depicting the approximate localization of the sfGFP insertion and the protein truncations caused by the PTC+ indel mutations. (**c**) Sum of confocal z-stack slices of the CNS of a third instar larva stained with anti-GFP (green) to show sfGFP::Lgr3 expression (green) and with anti-nc82 (magenta) and DAPI (blue) counterstains to show the synapses (neuropil) and nuclei, respectively. Arrows point to two bilateral pairs of PIL neurons (top), to the MIL neurons in the midline of the VNC (middle), and the distal VNC pair (bottom). Arrowheads point to the proximal projections of the PIL neurons. sfGFP::Lgr3 is also expressed in ∼170 other cell bodies, but at a lower level than in PIL and MIL neurons. f, oesophageal foramen, seg, subesophageal ganglion. See also Supplementary Fig. 4c for controls on the specificity of this staining pattern. (**d**) Box plots (see Methods) showing pupariation time of (*N*) larvae obtained from 11 egg layings. Whiskers are 5 and 95% percentiles. Dots, outliers. *P<*0.0001, Kruskal–Wallis test. Genotypes sharing the same letter (blue) are not statistically different at *α*=0.01, Conover *post hoc* test. Degrees of freedom, *H* and *C* values for the Kruskal–Wallis tests are df=7, *H*=397.84 and *C*=1.00. Scale bar, 50 μm.

To gain insight into the tissue and cellular expression pattern of *Lgr3* at the protein level, we used CRISPR/Cas9-mediated homologous repair^15^,^16^ to tag the endogenous Lgr3 protein at its amino terminus with superfolder green fluorescent protein (sfGFP^17^; Fig. 3a,b and Supplementary Fig. 4a). We obtained one allele, *Lgr3*^*ag5*^, hereafter named *sfGFP::Lgr3*, which contained an intact *Lgr3-*coding sequence downstream of the sfGFP insertion (Supplementary Fig. 4a). sfGFP::Lgr3 labels ∼180 CNS cell bodies, consisting of ∼40 cell bodies in the brain proper and ∼140 in the ventral nerve cord (VNC) of third instar larvae CNS (Fig. 3c). No other larval tissue apart from the CNS had detectable fluorescence or anti-GFP staining. This suggests that *Lgr3* does not cell-autonomously control ecdysone biosynthesis in the prothoracic gland downstream of Dilp8 (Supplementary Fig. 4b). The effectiveness of *sfGFP::Lgr3* as a protein translation reporter was further confirmed by analysing other *sfGFP* insertion alleles with PTC-inducing (PTC+) indels in the *Lgr3-*coding region (Supplementary Fig. 4a) or by using RNAi against endogenous *Lgr3*. Results show that either PTCs or RNAi against *Lgr3* effectively reduced sfGFP::Lgr3 expression to undetectable levels (Supplementary Fig. 4c, right panels), demonstrating the reliability of the *sfGFP::Lgr3* endogenous protein reporter and further certifying the effectiveness of the *Lgr3* RNAi. Importantly, PTC+, but not PTC− alleles suppressed the delay induced either by raising the larvae in the presence of the genotoxic agent ethylmethanosulfonate (EMS), which induces apoptosis, tissue damage/regeneration, an imaginal-disc-specific Dilp8 upregulation and consequentially a robust delay in the onset of metamorphosis^3^ (Fig. 3d), or by ectopically expressing Dilp8 (Supplementary Fig. 5). These results confirm that the PTC+ alleles are loss-of-function alleles and demonstrate that the PTC− *sfGFP::Lgr3 ag5* allele encodes a functional receptor. Together, these results strongly suggest that the Lgr3 protein acts in a subpopulation of CNS neurons.

**Figure 4:**
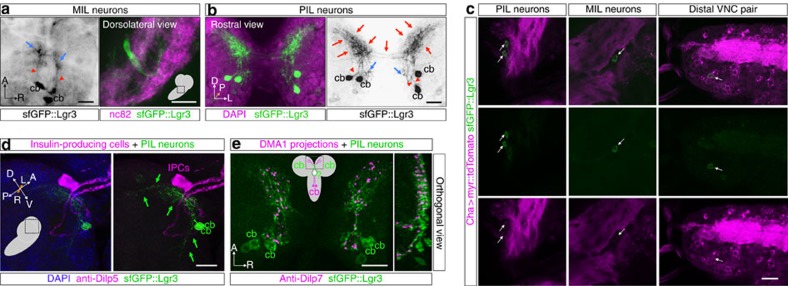
Neuroanatomy of neuronal populations strongly expressing Lgr3. (**a**) MIL neuron cell bodies (cb) located deep in the VNC project ventrally and anteriorly for a short distance (red arrowheads). Adjacent anterior projections are depicted (blue arrows). The left and right panels represent views of MIL neurons from different CNS preparations. The left panel is an inset of the sfGFP::Lgr3 channel of the CNS depicted in Fig. 3c. (**b**) Rostral view of the *pars intercerebralis* depicting the PIL neurons stained with anti-GFP (green (left) and black (right)) and counterstained with DAPI (magenta). Possible proximal dendritic arborizations are indicated with red arrows. Blue arrows indicate a ramification, likely axonic, with an undetermined terminus. Primary neurites (red arrowhead). (**c**) Overlap between *Cha>myr::tdTomato* and *sfGFP::Lgr3* expression patterns. Single confocal slices showing the neurons expressing highest levels of *Lgr3* (PIL, MIL and distal pair of VNC neurons, arrows in upper, middle and lower panels, respectively) labelled with sfGFP::Lgr3 (anti-GFP, green) and *Cha>* neurons were visualized with a *UAS-myr::tdTomato* reporter (magenta). (**d**) PIL neurons associate closely to IPCs. Z-stack projection of confocal stacks stained with anti-GFP (green) and anti-Dilp5 (magenta). (**e**) PIL neurons associate closely to Dilp7-producing DMA1 neurons in the *pars intercerebralis*. Z-stack projection and orthogonal view of confocal stacks stained with anti-GFP (green) and anti-Dilp7 (magenta). Scale bars, 20 μm.

We were unable to detect sfGFP::Lgr3 expression in neurons directly innervating the ring gland (Figs 3c and 4 and Supplementary Fig. 4b,d), suggesting that the Lgr3 neurons do not control developmental timing by direct cellular contact or synapsis with the ring gland, such as through PTTH-producing neurons^18^, the insulin-producing cells (IPCs)^19^ or subesophageal serotonergic neurons^20^, all of which have been shown to modulate developmental timing. This scenario suggests that the Lgr3-positive neurons represent a cellular population that has not been previously linked to growth and developmental timing control.

To gain insight into how Lgr3 neurons transmit the Dilp8 signal to the prothoracic gland, we studied the neuroanatomy and neurotransmitter profile of *sfGFP::Lgr3* neurons in more detail. sfGFP::Lgr3 expression was not homogeneous in the ∼180 CNS cell bodies. It was most strongly expressed in a single pair of neurons in the very tip of the VNC, in a single dorso-ventral pair of midline neurons located deep in the thoracic segment of the VNC (hereafter abbreviated to MIL neurons (midline internal Lgr3 neurons)), and in a pair of bilateral neurons localized in the anterior part of the *pars intercerebralis* (hereafter abbreviated to PIL neurons (*pars intercerebralis* Lgr3 neurons)) (Fig. 3c arrows; and Fig. 4a,b). All three neuronal populations express *sfGFP::Lgr3* and present their characteristic neuroanatomy already at the L1 stage (Supplementary Fig. 6). Co-staining of *sfGFP::Lgr3* with *choline acetyltransferase (Cha)-Gal4* driving a *UAS-myr::tdTomato* reporter indicates that many *Lgr3-*positive neurons are cholinergic, including all three major neuronal populations (PILs, MILs and the pair of distal VNC neurons (Fig. 4c)). Co-staining with *glutamic acid decarboxylase (Gad1)-Gal4* and *Vesicular glutamate transporter promoter* (*VGlut)-Gal4*, which drive expression in GABAergic and glutamatergic neurons, respectively, label very faintly the PIL neurons and the pair of distal VNC neurons and show no detectable staining in MIL neurons (Supplementary Fig. 7a,b). MIL neurons project their neurites ventrally and then anteriorly towards the brain, branching close to the base of the brain (Fig. 4a). PIL neurons extend a single neurite centripetally into the neuropil that branches ventrally towards the subesophageal region and dorsally into highly arborized proximal termini (Fig. 4b). The latter reach the nearby IPC bodies from immediately underneath, while the IPC neurites extend through the PIL neuron arborization (Fig. 4d). PIL neurons also show an intimate association with the axonal projections of the Dilp7-producing dorsal medial (DMA1)^21^ (Fig. 4e). These results provide anatomical and possible functional context for an anterograde neuronal input for PIL neurons (considering that proximal branches are typically dendritic)^22^.

### Lgr3 acts in the CNS to delay development

**Figure 5:**
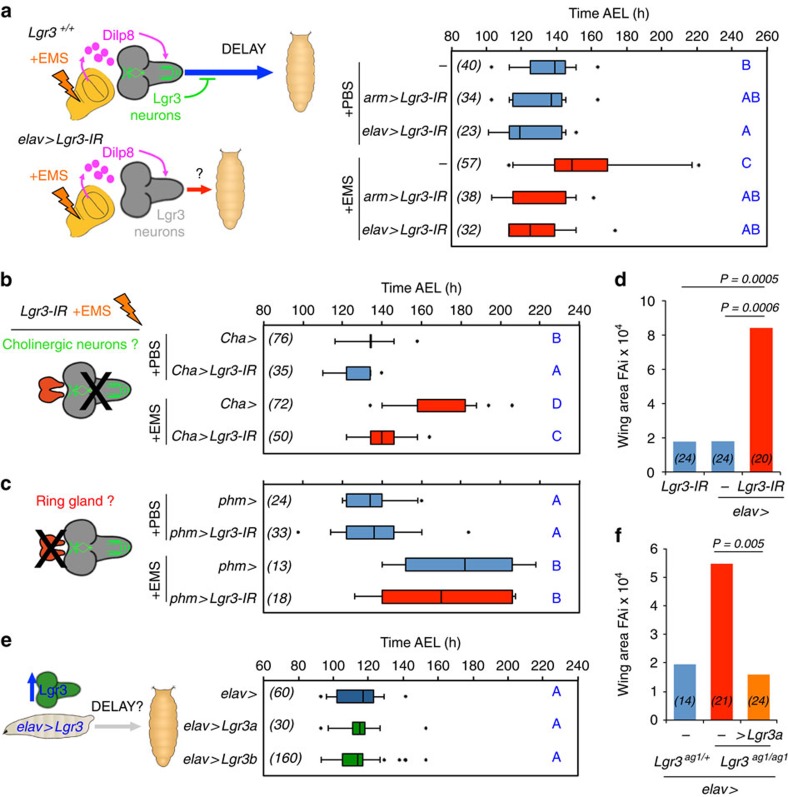
Lgr3 is required in cholinergic neurons to couple growth and developmental timing. (**a**) CNS-specific RNAi of *Lgr3* rescues the EMS-induced delay. (**b**) Removal of *Lgr3* in cholinergic neurons (using *Cha>*) rescues the EMS-induced delay. (**c**) Ring gland-specific RNAi of *Lgr3* does not significantly affect pupariation time in EMS assays. (**d**) FAi in (*N*) animals expressing RNAi against *Lgr3* in neurons using *elav>* driver (F-test). df_(*elav>Lgr3-IR*)_=19, df_(*Lgr3-IR*)_=23, F=4.71 and df_(*elav>Lgr3-IR*)_=19, df_(*elav>*)_=23, F=4.65. (**e**) Ectopic expression of *Lgr3* is not sufficient to delay the onset of metamorphosis. Two *UAS-Lgr3* transgene insertions (*Lgr3a* and *Lgr3b*) were tested. (**f**) FAi in (*N*) animals carrying mutations in *Lgr3* and rescued with neuronal expression of *Lgr3a*. df_(elav>;Lgr3(ag1))_=20, df_(elav>Lgr3a;Lgr3-IR)_=23, F=3.41; df_(elav>;Lgr3(ag1))_=20, df_(elav>Lgr3(ag1/+)_=13, F=2.88. (**a**–**c**,**e**) Box plots (see Methods) showing pupariation time of (*N*) larvae obtained from six, two, four and six egg layings for **a**–**c**,**e**, respectively. Whiskers are 5 and 95% percentiles. Dots, outliers. *P<*0.0001, Kruskal–Wallis test for all panels, except for **e** where *P=*0.0413. Genotypes sharing the same letter (blue) are not statistically different at *α*=0.01, Conover *post hoc* test. Degrees of freedom, *H* and *C* values for the Kruskal–Wallis tests are df=5, *H*=55.06, *C*=0.99; df=3, *H*=150.64, *C*=0.99; df=3, *H*=37.31, *C*=0.99; df=2, *H*=6.32, *C*=0.90, for **a**–**c**,**e**, respectively.

The sfGFP::Lgr3 expression pattern suggests that disrupting receptor function exclusively in the CNS should be sufficient to suppress the Dilp8-dependent delay. To test this, we crossed the *Lgr3-IR-V22* RNAi line to the panneuronal driver *elav-Gal4* (*elav>*) and performed EMS assays (Fig. 5a). Reducing *Lgr3* function in *elav>* cells leads to complete suppression of the EMS-induced delay, as also observed with ubiquitous knockdown using *arm>* (Fig. 5a). The neuronal requirement of *Lgr3* was further confirmed by knockdown of *Lgr3* in *elav* cells in a context of ectopic expression of *dilp8* (Supplementary Fig. 8a), and by rescuing neuronal expression of *Lgr3* in an *arm>dilp8 Lgr3-IR-V22* context by inhibiting *Lgr3* RNAi exclusively in the CNS by expressing the Gal4 inhibitor, Gal80, under the control of the *elav* enhancer (*elav-Gal80*; Supplementary Fig. 8b). As expected from our neuroanatomical and neurotransmitter biosynthesis expression pattern studies, knockdown of *Lgr3* in cholinergic neurons using *Cha-Gal4* also significantly suppressed the EMS-induced delay (Fig. 5b). In contrast, knockdown of *Lgr3* in the ring gland using *phantom (phm)-Gal4* did not rescue the EMS-induced delay (Fig. 5c). If *Lgr3* is required in the CNS for the Dilp8-dependent delay, then its removal in the CNS alone should mimic the increased FAi phenotype of *Lgr3* mutants. Consistently, the FAi of *elav>Lgr3-IR-V22* adults was increased by an order of 4 when compared with *elav>* controls (*P=*0.0005, F-test; Fig. 5d). We conclude that *Lgr3* is required in the CNS, likely in one or more of the ∼180 Lgr3-positive cholinergic neurons described above, to convey the Dilp8-dependent developmental delay.

According to heterologous studies in human cell lines *in vitro*^10^, *Lgr3* is a constitutively active receptor. If this were true *in vivo* and if Dilp8 activity would inhibit Lgr3 activity, we would expect that by removing Lgr3 activity we would see a delay, which is not the case (see controls in Figs 2a, 3d and 5a). To look at the effect of removing Lgr3 activity on pupariation timing at an increased resolution, we resynchronized larvae at the second to the third instar molt and confirmed that *Lgr3* mutants do not have a delayed development, but rather pupariate ∼4 h earlier than controls, independent of their genetic background (Supplementary Fig. 9). Even though our loss-of-function studies do not rule out constitutive activity, they are more consistent with a scenario where Lgr3 is activated either by Dilp8 or by other downstream Dilp8-dependent signals. We thus hypothesized increased Lgr3 levels would not lead to a delay. Alternatively, if Lgr3 were somehow constitutively active, it would delay pupariation timing if ectopically expressed. To conduct this experiment, we constructed a *UASP-Lgr3* transgene and expressed it in the CNS under the control of *elav>*. We find that the pupariation time profile of *elav>Lgr3* animals is indistinguishable from controls (Fig. 5e). Control experiments suggest that the *UASP-Lgr3* transgene carries functional Lgr3 activity, as it rescues the FAi of *ag1* animals when driven by *elav>* (Fig. 5f). These results argue against a simple explanation where Lgr3 is constitutively active^10^. Instead, they are in line with our findings that, in the absence of tissue growth aberrations or ectopic Dilp8 expression, Lgr3 activity does not have a major impact on timing the onset of metamorphosis. These results are coherent with a model where Lgr3 is activated either directly or indirectly by the Dilp8 signal and hint towards the existence of a dedicated tissue stress-sensing pathway in *Drosophila*.

### Lgr3 neurons relay the Dilp8 delay signal

**Figure 6:**
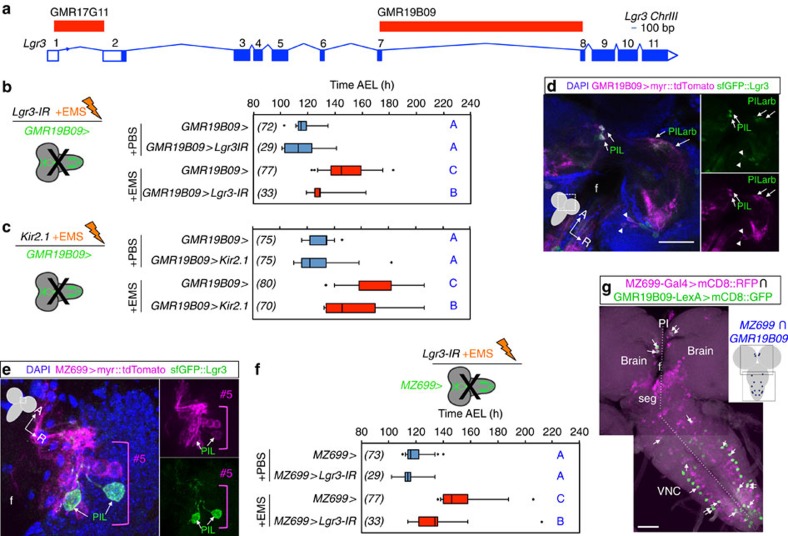
A restricted subset of Lgr3 neurons relays the Dilp8 signal to the ring gland. (**a**) Scheme of the *Lgr3* locus depicting the regulatory elements^23^,^24^ tested in this study. (**b**) RNAi of *Lgr3* in *GMR19B09>* neurons rescues the EMS-induced delay. (**c**) Silencing of *GMR19B09>* neurons by Kir2.1 expression suppresses the EMS-induced delay. (**d**) *GMR19B09>* (magenta) drives expression in sfGFP::Lgr3-positive neurons (green): PIL neurons (PIL, arrows); PIL proximal arborizations (PILarb, arrows) and other neurons (arrowheads). (**e**) PIL neurons (green) are a subset of #5 neurons (magenta) defined by *MZ699>*. (**f**) RNAi of Lgr3 in *MZ699>* neurons rescues the EMS-induced delay. (**g**) Intersectional pattern of *GMR19B09-LexA* driving *lexAop-mCD8::GFP* (green) and *MZ699>mCD8::RFP* (magenta). Cells expressing both drivers are depicted with arrows in the two overlapping max-intensity projections of confocal z-stack sections and as blue dots in the CNS cartoon to the right. (**f**) oesophageal foramen. seg, subesophageal ganglion. The midline is depicted with a dashed line. A more detailed image of the PIL neurons in separate green and magenta channels is available in Supplementary Fig. 13. (**b**,**c**,**f**) Box plots (see Methods) showing pupariation time of (*N*) larvae obtained from two egg layings for each panel. Whiskers are 5 and 95% percentiles. Dots, outliers. *P<*0.0001, Kruskal–Wallis test for all panels. Genotypes sharing the same letter (blue) are not statistically different at *α*=0.01, Conover *post hoc* test. Degrees of freedom, *H* and *C* values for the Kruskal–Wallis tests are df=3, *H*=83.48, *C*=1; df=3, *H*=177.75, *C*=0.97; df=3, *H*=156.71, *C*=0.99, for **b**,**c**,**f**, respectively. Scale bars, 50 μm (**d**,**g**); 20 μm (**e**).

To further narrow down the identity of the neurons requiring *Lgr3*, we tested the ability of two Janelia Gal4 lines^23^ carrying regulatory regions of the *Lgr3* locus, *GMR17G11-Gal4 (GMR17G11>)* and *GMR19B09-Gal4 (GMR19B09>)* (Fig. 6a), selected based on their larval brain expression patterns^24^, to suppress the EMS-induced developmental delay when driving *Lgr3-IR-V22.* While *GMR17G11>Lgr3-IR-V22* had no effect on the EMS-delay (Supplementary Fig. 10a), the *GMR19B09>Lgr3-IR-V22* condition significantly reduced the 31.6-h EMS-dependent delay in pupariation time to 15.7 h (∼50% reduction, *P<0.01,* Conover *post hoc* test; Fig. 6b). We conclude that the subset of neurons expressing *GMR19B09>* is critical for the Dilp8 and Lgr3-dependent coupling of growth and developmental timing. Silencing of these same neurons by expression of the inward rectifying K+ channel (Kir2.1) throughout embryonic and larval development was compatible with developmental progression and led to a significant suppression of the *EMS-*dependent delay (*P<0.01*, Conover *post hoc* test (Fig. 6c). These results suggest that the activity of *GMR19B09>* neurons dictates the central Dilp8 and Lgr3-dependent response to tissue damage.

The *GMR19B09>* driver labels ∼270 neurons, ∼30 of which populate each brain hemisphere^24^. By labelling *GMR19B09>* neurons with *myr::tdTomato* in a *sfGFP::Lgr3* background, we detect overlapping expression in ∼10 neurons per hemisphere, 2 of which are the bright PIL neurons (Fig. 6d). In contrast, *GMR17G11>*, which does not suppress the Dilp8-dependent delay, is not expressed in the bright PIL neurons^24^ (Supplementary Fig. 10b). We find no detectable *GMR19B09>myr::tdTomato* expression in MIL neurons and only a very faint trace, if any, in the posterior distal VNC pair (Supplementary Fig. 11a,b). Of the ∼200 *GMR19B09-*positive VNC cells, roughly 60 co-express *sfGFP::Lgr3* at low, yet detectable, levels. Any of the ∼70 CNS neurons coexpressing *sfGFP::Lgr3* and *GMR19B09>* are good candidate cells to convey the Dilp8 signal.

Since *GMR19B09>*, but not *GMR17G11>*, clearly colocalizes with PIL neurons, one of the cell populations that most strongly expresses *Lgr3,* we looked for other Gal4 lines that could allow the genetic manipulation of these neurons. The *MZ-699-Gal4* (*MZ699>*) line drives expression in similar type of neurons, named #5 neurons^25^. Like PIL neurons, #5 neurons contribute to projections at the anteriormost region of the brain neuropil and to the median bundle tract that follows the oesophageal foramen^25^*. MZ699>myr::tdTomato* expression analysis in a *sfGFP::Lgr3* background demonstrates that PIL neurons are a subset of #5 neurons (Fig. 6e). To test whether silencing *Lgr3* in *MZ699>* cells could rescue the tissue-damage-dependent developmental delay, we drove *Lgr3-IR-V22* under the control of *MZ699>* and exposed the larvae to EMS. We find that *MZ699>Lgr3-IR-V22* strongly suppresses the EMS-dependent delay (Fig. 6f). Apart from #5 neurons, colocalization of both transgenes in the brain region was only detected in three other neurons that appear to be a subset of #2 neurons^25^. However, *GMR19B09>* does not appear to label these cells, making it unlikely that they contribute to the Lgr3-dependent Dilp8 signal transduction. Other subesophageal neurons and many other VNC neurons, with the notable exception of the strong distal VNC neuronal pair, express both *MZ699>myr::tdTomato* and *sfGFP::Lgr3* (Supplementary Fig. 12a,b). As *GMR19B09*> labelling of the subesophageal region is sparce^24^, and neither *MZ699>* or *GMR19B09>* drives detectable expression in MIL neurons (Supplementary Figs 11a and 12a, respectively), this suggests that either the PIL neuron expression overlap could be functionally relevant or that the Dilp8 signal requires Lgr3 expression in any of the ∼60 *sfGFP::Lgr3-*positive VNC cells.

To further narrow down the Lgr3-positive neurons required for Dilp8 activity, we analysed the intersectional expression between the *GMR19B09* and *MZ699* drivers. For this, we labelled the *MZ699>* neurons with a *UAS-mCD8::RFP* transgene and the *GMR19B09* neurons using a *GMR19B09-LexA*-driven *lexAop-mCD8::GFP* transgene^24^,^24^. Consistent with our findings above, only the two bilateral PIL neurons showed overlapping expression of *GMR19B09* and *MZ699* in the brain (Fig. 6g and Supplementary Fig. 13). In the VNC, we found overlap in seven bilateral neurons (Fig. 6g). These data limit the requirement of Lgr3 in the Dilp8 pathway to eight bilateral neuronal populations.

### Dilp8 activates cAMP signalling in PIL neurons

**Figure 7:**
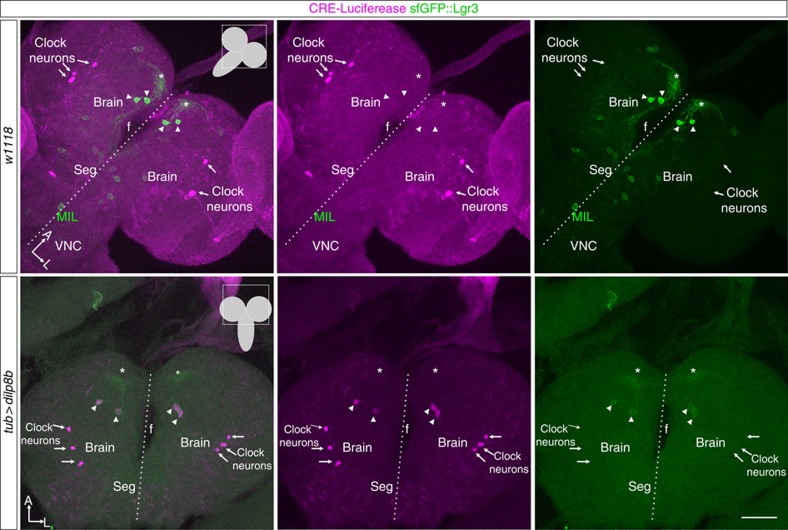
Dilp8 activates cAMP signaling in PIL neurons, but not other Lgr3-positive neurons. Maximum intensity projections of confocal z-stack slices of CNS preparations of larvae carrying a *CRE-luciferase* reporter (anti-luciferase, magenta) and *sfGFP::Lgr3* (anti-GFP, green) in a control background (*w*^*1118*^, upper panels) or following constitutive ectopic Dilp8 expression (*tub>dilp8b* background, lower panels). PIL neuron cell bodies (arrowheads) do not express detectable CRE-luciferase (anti-luciferase, magenta), except when activated by Dilp8 expression. Anti-luciferase staining in clock neurons (arrows) serves as an internal control. Background anti-luciferase staining in glial cells is also detectable throughout the CNS. f, oesophageal foramen. Asterisk labels PIL neuron proximal arborizations. Midline is labelled with a dashed line. Scale bar, 50 μm.

Lgr3 has been shown to constitutively activate cyclic AMP (cAMP) in heterologous studies in human cell lines *in vitro*^7^. To study whether or not Dilp8 could modulate cAMP activity *in vivo*, we ectopically expressed Dilp8 using *tub>* in larvae carrying a cAMP response element (CRE)-(FRTmCherry) luciferase reporter^26^, and followed CRE-dependent expression of both mCherry and luciferase in the third instar larval CNS by immunofluorescence using an antibody against luciferase in the red channel (hereafter, anti-luciferase). In control preparations, significant anti-luciferase expression is detected in the CNS in bilateral neurons resembling PDF-positive clock neurons and in glial cells throughout the CNS (Fig. 7, upper panels). Dilp8 expression led to a detectable anti-luciferase staining in an additional cell population, the PIL neurons in the brain, as determined by co-staining between anti-luciferase and anti-GFP in brains carrying both the *CRE-luciferase* and the *sfGFP::Lgr3* reporters (Fig. 7, lower panels). Importantly, no luciferase expression was detected in MIL neurons or other brain neurons that express *sfGFP::Lgr3*, despite the ectopic constitutive Dilp8 expression in every cell (Supplementary Fig. 14a,b). We conclude that PIL neurons respond to Dilp8 by increasing cAMP levels.

### Candidate cell surface receptors for Dilp8

**Figure 8:**
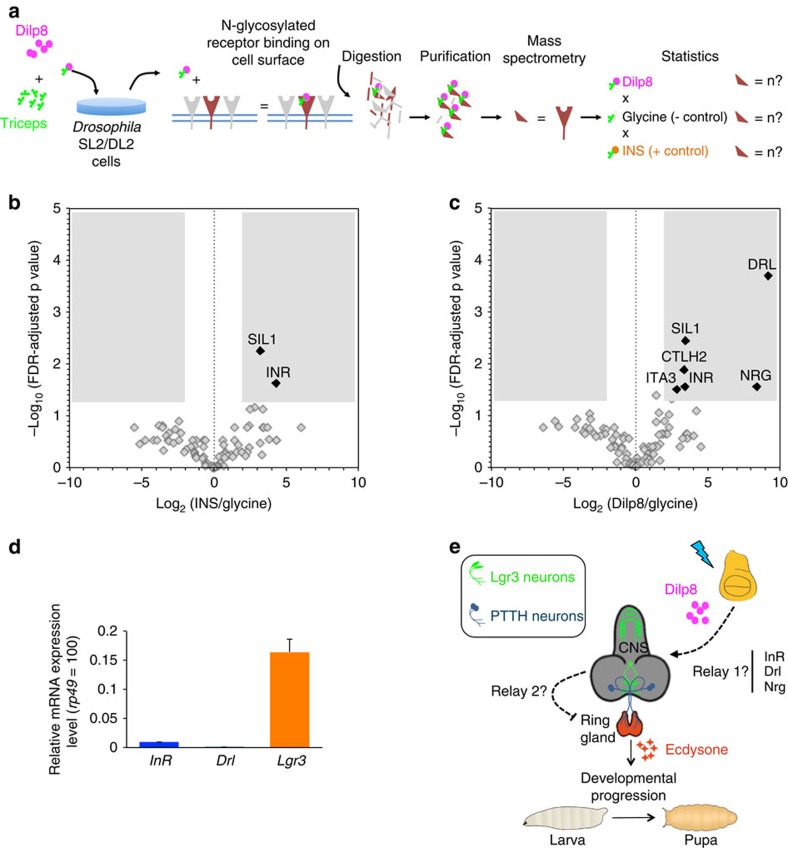
Determination of candidate cell surface receptors for Dilp8 and a multiple relay model of the Dilp8–Lgr3 developmental stability pathway. (**a**) Scheme of the ligand–receptor capture assay (LRC). TriCEPS reagent coupled with Dilp8 (or with human insulin and glycine as positive and negative controls, respectively) was assayed in triplicate in *Drosophila* SL2/DL2 cells. (**b**) Volcano plots (false-discovery rate (FDR)-adjusted *P* values plotted against the fold changes (FC) between samples) comparing control TriCEPS-bound human insulin ligand against the glycine-quenched TriCEPS reagent control sample. The shaded area represents the receptor candidate space, defined by an enrichment factor greater than 4 and an FDR-adjusted *P<*0.05. (**c**) Volcano plots comparing TriCEPS-bound Dilp8 ligand against the glycine-quenched TriCEPS reagent control sample. The shaded area represents the receptor candidate space, defined by an enrichment factor of greater than a factor of 4 and an FDR-adjusted *P<*0.05. (**d**) Relative mRNA levels of different receptor genes in *Drosophila* SL2/DL2 cell line. Values represent the geometric mean±s.e.m. (*n*=3 biological repeats, except for *Drl*, where two biological repeats were made) of *InR, Drl* or *Lgr3* mRNA levels relative to *rp49* levels (*rp49=*100). (**e**) Cartoon depicting the Dilp8–Lgr3 abnormal tissue growth-sensing pathway. Dilp8 either signals directly onto Lgr3 neurons located in the CNS in a process where any of the other candidate direct Dilp8-binding proteins can act as a co-factor to Lgr3, or those receptors/co-receptors could play a role in relaying the Dilp8 signal from the periphery to the Lgr3 neurons in the CNS (Relay 1). The second Relay (Relay 2) would occur from the Lgr3-positive neurons to the ring gland.

Our data suggest that Lgr3, a member of the conserved relaxin receptor-like family, is an essential component of the pathway that couples growth to developmental timing control via the peptide hormone Dilp8. Lgr3 can act as a direct receptor for Dilp8 or alternatively as an intermediate factor in the Dilp8 pathway. To start gaining insight into these two possibilities, we carried out unbiased ligand–receptor capture (LRC) assays^27^,^28^ using mature Dilp8 (ref. 29) as a ligand and *Drosophila* SL2/DL2 cells. In this assay, Dilp8 is covalently attached to TriCEPS^27^,^28^ reagent, which allows capture of N-glycosylated cell surface proteins from gently oxidized live cells and the subsequent biotin-based purification of captured glycopeptides for mass spectrometry analyses^27^,^28^ (Fig. 8a). Statistical analyses of the results of the control experiments with glycine-quenched TriCEPS reagent and human insulin (which is known to bind and activate the sole *Drosophila* insulin-like receptor (InR)^30^) as negative and positive controls, respectively, showed the consistent detection of the InR in the insulin-bound fraction, as expected^30^, demonstrating the successful application of the LRC assay in *Drosophila* cells (Fig. 8b and Supplementary Tables 1 and 2). An endoplasmic reticulum resident protein, the nucleotide exchange factor SIL1, was also enriched in the insulin-bound fraction. SIL1 has been recently linked to insulin secretion in mice, suggesting that this direct link to insulin should be further explored^31^.

Similar to the human insulin, Dilp8 captured the InR and SIL1, but in addition it also captured four other candidate binding proteins: the receptor Tyrosine kinase (RYK)-like factor derailed (Drl), neuroglian (Nrg), the laminin receptor integrin α-PS3 (ITA3) and the choline transporter (CTL)-like protein 2 (*CTLH2*) (Fig. 8c, Supplementary Tables 1 and 2). Any of these proteins can function as a direct receptor or co-receptor for Dilp8. The InR and Drl receptors are otherwise thought to function as highly conserved receptors for the insulin and Wnt5 signalling peptides, respectively, in flies and humans, which have functions in growth and axonal pathfinding^32^, respectively. Importantly, no Lgr3 was obtained in these experiments, even though SL2/DL2 cells express considerable levels of Lgr3 mRNA (higher than InR by a factor of ∼20; Fig. 8d). While these data do not exclude a direct ligand–receptor interaction between Dilp8 and Lgr3, they demonstrate that Dilp8 can directly bind to the InR, an expected candidate receptor for an Ilp-family member such as Dilp8.

## Discussion

Different organs need to sense growth perturbations in distant tissues to coordinate their size and differentiation status during development^1^. Here we have determined that the sensing of peripheral growth perturbations requires a novel population of CNS neurons expressing the Lgr3 relaxin receptor. Neuronal Lgr3 is required for the transmission of the peripheral growth aberration signal, Dilp8, to the prothoracic gland, which controls the onset of metamorphosis and thereby the cessation of imaginal disc growth^1^,^3^,^4^,^5^,^6^,^7^,^12^,^33^ (Fig. 8e). This work reveals a new Dilp8–Lgr3 pathway that is critical to ensure developmental stability in *Drosophila*. Our study opens many questions for further research, such as the determination of which of the eight bilateral Lgr3-positive interneuron populations (Fig. 6g) are critical during Dilp8 expression, whether or not the interaction between Lgr3 and Dilp8 is direct and how Lgr3-positive neurons relay information to the ring gland.

Of the eight bilateral Lgr3-positive interneuron populations identified in our study, the cholinergic PIL neurons both require *Lgr3* for the Dilp8-dependent developmental delay activity and respond to Dilp8 by increasing cAMP levels. Therefore, PIL neurons are the best candidates to mediate the Dilp8-dependent developmental delay. Further research is necessary to determine if PIL neurons are sufficient to regulate developmental timing in the absence of growth aberrations or ectopic Dilp8 signals.

While our results clearly indicate that Dilp8 and Lgr3 act on the same pathway, their biochemical relationship is less clear. As Dilp8 is an Ilp and Lgr3 is a homologue of a vertebrate receptor for an Ilp (relaxin), it is tempting to propose a direct ligand–receptor interaction between them. This possibility is supported by the strong genetic interaction between *dilp8* and *Lgr3* and our finding of Dilp8-responsive Lgr3-positive neurons. However, our study also raises at least three possible issues with this interpretation of our data. First, the neuroanatomy of the CNS neuronal populations requiring Lgr3 activity suggests that Dilp8 could have to traverse the blood–brain barrier to activate Lgr3-positive interneurons deep in the brain, something which is presently unclear if it can be achieved. Alternatively, our data can also be explained if the Dilp8 signal is received by other cells (if by the CNS, these can be either glial cells or other neurons with projections exposed to the haemolymph), and relayed through one or more steps before reaching the Lgr3-positive cells (Fig. 8e, ‘Relay1?'). A similar route through blood–brain barrier glial cells has been proposed to explain the relay into the CNS of a fat-body-derived signal that controls neuroblast reactivation^34^,^35^,^36^.

Second, we have failed to identify Lgr3 among candidate Dilp8-binding cell surface receptors/co-receptors. Clearly, the biochemical identification of alternative cell surface-binding proteins such as the InR, Nrg and the RYK-like Drl^37^,^38^ does not rule out the possibility of a direct interaction between Dilp8 and Lgr3 *in vivo*, nevertheless it strongly indicates that Dilp8 can consistently interact with a likewise strong receptor candidate for an Ilp, such as the InR. The LRC technique we used can identify receptors of interest with affinities spanning 4 orders of magnitude at expression levels as low as 2,000 receptors per cell (www.dualsystems.com). However, it is not yet clear how quantitative it can be relative to affinity constants. Dilp8 has been previously shown to modulate growth *in vivo* often in opposite ways depending on the observed tissue^3^. Namely, Dilp8 ectopic expression throughout development leads to heavier adults and to reduced expression of the translational inhibitor and FOXO-target *Thor* (*4E-BP*) in the larval fat body, which is consistent with a local increase in insulin/IGF-like signalling^3^. In contrast, *Thor* levels are higher in imaginal discs in the same animals^3^. These results show that understanding the relationship between Dilp8, InR and Lgr3 will be a challenge for further studies. One possibility, if Dilp8 can indeed interact with Lgr3 in other contexts, is that there is a crosstalk between Lgr3 and InR receptors. The other possibility is that Dilp8 has a low-affinity interaction with the InR, which could be potentiated in certain physiological conditions. Affinity profiling of the Dilp8 and InR interaction, as well as that of Lgr3, should bring insight into this scenario. As regards the other Dilp8-specific candidate receptor, Drl, it also binds to Wnt5 (ref. 32) to control aspects of axonal guidance, raising the possibility that the interaction between Dilp8 and Drl, if confirmed, can interfere with circuit formation. Interestingly, Drl has been shown to be expressed in four large glial cells in the interhemispheric region of the brain, close to the PIL neurons, and to be dynamically regulated between the third instar larvae and early pupae^37^. Therefore, the interaction between Dilp8 and Drl should be carefully followed-up and independently verified.

Third, the fact that ectopic expression of Dilp8 only leads to a detectable increase in cAMP signalling in PIL neurons, and not in other Lgr3-positive neurons (Fig. 7), indicates that Lgr3 activation by Dilp8 requires other molecular and/or cellular players. Any of the factors identified biochemically in this study could participate in PIL neuron selectivity, for instance, as a differentially enriched co-receptor. Alternatively, PIL neurons could be selectively activated downstream of other cellular players, via a mechanism which could involve a signal relay by direct synapsis or proximity to other cells that participate in the transduction of the Dilp8 signal from the periphery to the ring gland. In this case, Dilp8 would probably activate Lgr3-positive neurons indirectly. Therefore, in the absence of further evidence suggesting a direct relationship between Dilp8 and Lgr3, we cannot rule out the possibility that Lgr3-positive neurons are not a direct target of Dilp8, but rather intermediary players in the Dilp8 developmental stability pathway.

How the Dilp8 signal reaches the ring gland after having triggered activity in some of the eight bilateral Lgr3-positive neuronal groups remains to be determined. The fact that we were unable to detect *sfGFP::Lgr3* or *GMR19B09>myr::tdTomato* expression in the ring gland or in neurons innervating the ring gland (Figs 3c and 4 and Supplementary Fig. 4b,d), strongly suggests that the Lgr3-positive neurons that are required for the Dilp8-dependent delay do not connect directly to the ring gland. Hence, it is likely that the Lgr3 neurons also need to relay the tissue stress signal at least once to the ring gland, either by secreting a second factor or connecting to a ring gland-innervating neuron (Fig. 8e, ‘Relay2?'). Together, these results indicate that the peripheral Dilp8 tissue damage signal is transduced through multiple steps before it reaches the ring gland, revealing unprecedented complexity and providing both important functional insight into the transduction of the Dilp8-dependent aberrant tissue growth signalling pathway and opening fertile ground for further research.

The similarities between the neuroendocrine mechanisms controlling the larval-to-pupal transition in *Drosophila* and the hypothalamic-pituitary axis in vertebrates has been highlighted^2^,^39^. The neurosecretory cell-rich *pars intercerebralis*, in which the Dilp8-responding and Lgr3-expressing PIL neurons are located, has anatomical, developmental and functional analogies to the hypothalamus, the structure that integrates the vertebrate CNS to the endocrine system via the pituitary gland. Similarly, the *Drosophila pars intercerebralis* connects the CNS to the endocrine ring gland complex via neurosecretory cells^40^, such as the IPCs^19^,^40^. Both systems have roles in stress response, energy metabolism, growth, water retention and reproduction^8^,^39^. The neuroanatomy of Lgr3-positive neurons, such as the PIL neurons (Figs 3c and 4b), suggests they are well-positioned to relay signals or to modulate the activity of ring gland-innervating neurons during tissue stress events that trigger Dilp8 secretion from the periphery. Candidate neurons that could interact with PIL neurons are the IPCs, PTTH neurons and DMA1 neurons. Apart from arborizing in the *pars intercerebralis* region (Figs 3c and 4b), PIL neurons send projections via the median bundle to the subesophageal region^24^,^25^. This region is known to harbour the serotonergic SE0-PG neurons, which directly innervate the PG, thereby regulating developmental timing as a response to nutritional cues^20^. It will be interesting to test whether PIL and SE0-PG neurons synapse in the subesophageal region and whether the latter also have a role in the tissue damage response.

As the timing of vertebrate developmental transitions, such as puberty, can also be altered by intrinsic and extrinsic factors affecting body growth, such as inflammatory disease and nutritional status^2^, the exploration of the role of relaxin signalling in modulating the hypothalamic-pituitary axis is a promising area for research. This is highlighted by the fact that the hypothalamus expresses relaxin receptors, including the Lgr3-homologue, RXFP1, in mammals and fish^8^,^41^, suggesting that a central neuroendocrine role for relaxin receptors might have evolved before the vertebrate and invertebrate split. A candidate peptide to regulate hypothalamic-pituitary stress-responses via relaxin receptors is the neuropeptide Relaxin-3 (RLN3), which has been traditionally viewed as being the ancestor ligand for all vertebrate relaxins^42^,^43^. RLN3 is strongly expressed in stress-responsive neurons from the nucleus incertus that directly innervate and modulate hypothalamic activity^8^,^44^,^45^,^46^,^47^. Our results therefore reveal an unexpected and striking similarity between the Dilp8–Lgr3 pathway and the vertebrate relaxin signalling pathway and hint to an ancient stress-responsive pathway coordinating animal growth and maturation timing.

## Methods

### Stocks

The *Drosophila melanogaster* stocks w^1118^ Bx-gal4 (w^1118^ P{GawB}Bx^MS1096^), UAS-rpr (w^1118^; P{UAS-rpr.C}14), Lgr3-IR-V10 (y^1^ v^1^;{TRiP.JF03217}attP2), Lgr3-IR-V22 (y^1^ sc* v^1^; {TRiP.GL01056}attP2/TM3 Sb^1^), elav-Gal4 (P{w[+mW.hs]=GawB}elav^C155^), elav-Gal4 (P{w[+mC]=GAL4-elav.L}2/CyO), w^1118^;Mi{ET1}Lgr3^MB06848^, w^1118^; sna^Sco^/SM6a, P{w[+mC]=hsILMiT}2.4, tub-Gal4 (y^1^ w*; P{tubP-GAL4}LL7/TM3, Sb[1]), w^1118^; Lgr3^Df(3)BSC321^/TM6C Sb^1^ cu^1^, Gad1-Gal4 (P{w[+mC]=Gad1-GAL4.3.098}2/CyO), Cha-Gal4 (w^1118^; P{w[+mC]=Cha-GAL4.7.4}19B/CyO P{ry[+t7.2]=sevRas1.V12}FK1), GMR19B09-Gal4 (w^1118^; P{y[+t7.7] w[+mC]=GMR19B09-GAL4}attP2), GMR17G11-Gal4 (w[1118]; P{y[+t7.7] w[+mC]=GMR17G11-GAL4}attP2), y^1^ w* P{y[+t7.7] w[+mC]=10XUAS-IVS-mCD8::RFP}attP18 P{y[+t7.7] w[+mC]=13XLexAop2-mCD8::GFP}su(Hw)attP8, and w^1118^; P{y[+t7.7] w[+mC]=GMR19B09-lexA}attP40 were obtained from the Bloomington *Drosophila* Stock Center at Indiana University. The stock y^1^ M{w[+mC]=Act5C-Cas9.P}ZH-2A w* (reference *#16*) was obtained from Bestgene. The stock arm-gal4 (w*; P{w[+mW.hs]=GAL4-arm.S}11) was a gift from P. Domingos. The stocks *UAS-dilp8b::3XFLAG and UAS-dilp8c::3XFLAG* (reference #3) were a gift from M. Dominguez. The stock w*; P{GawB}Mz699 and UAS-myr::tdTomato/CyO; TM2/TM6B were a gift from M.L. Vasconcelos. Balanced lines were generated by crosses to the stock w^1118^; *If/CyO; MKRS/TM6B*, which was a gift from A. Jacinto. The stock *w; CRE-F-luc (II)* was a gift from J.C. Yin. The stock *y w; ptth-HA* was a gift from M. O'Connor. Stocks are maintained at low densities at 18 °C in a 12-h light/dark cycle.

### Generation of the *Lgr3* ^*ag1*^ allele

We generated a mutation in the *Drosophila Lgr3* locus by using the MiET1 transposase to remobilize the MB Minos element *Lgr3*^*MB06848*^ (reference #11), which is inserted in the seventh *Lgr3* intron, <100 bp from exon 7 (Fig. 1b and Supplementary Fig. 1a). Virgin *Lgr3*^*MB06848*^ females were crossed with males carrying the heat shock-inducible MiET1 transposase on a *Cy* balancer chromosome (*w*^*1118*^*; If/Cy-Minos; MKRS/TM6B*) and transferred to new vials every day^8^. After 48 h of development and until pupariation, the F1 progeny was given a 1-h 37 °C heat shock daily to induce the expression of the MiET1 transposase. *Cy-Minos/+; Lgr3*^*MB06848*^*/MKRS* or *TM6B* male adults were selected and individually crossed to the balancer strain *w*^*1118*^*; If/CyO; MKRS/TM6B*. A single eGFP-negative (lacking the *Mi{ET1}* element) male was selected from each cross and mated with *w*^*1118*^*; If/CyO; MKRS/TM6B* females. The putative *Lgr3* excisions were balanced over *TM6B* to obtain the following genotypes *w*^*1118*^*; +/+; Lgr3*^*MB06848*^ *excision/TM6B*. We obtained one imprecise excision that generated the *Lgr3*^*ag1*^ mutant allele and three precise excisions named *Lgr3*^*ag2*^*, Lgr3*^*ag3*^ and *Lgr3*^*ag4*^ all of which behaved the same way. In this study, the *Lgr3*^*ag2*^ line was used as the genetic background control for the imprecise excision allele *Lgr3*^*ag1*^.

To molecularly characterize the *Lgr3*^*ag1*^ deletion, we performed a series of PCR assays with primer pairs located around the *Lgr3*^*MB06848*^ insertion (Supplementary Fig. 1a). *Lgr3*^*ag1*^ was initially detected by the failure to amplify a 866-bp PCR product flanking the *Lgr3*^*MB06848*^ insertion (green arrows, Supplementary Fig. 1a), and then a 240-bp PCR product using a pair of primers on exon 8 and 9 to the right of the *Lgr3*^*MB06848*^ element (red arrows, Supplementary Figs 1a,b; gel images have been cropped for presentation. Full size images are presented in Supplementary Fig. 15), indicating that a deletion occurred downstream of the element following its remobilization. A PCR using a primer pair in the exon 11 produced a positive result, indicating that exon 11 was present in *Lgr3*^*ag1*^ (not shown). This suggested that while the *Lgr3*^*ag1*^ deletion was very large, its breakpoints were confined within the *Lgr3* locus. We then tried to amplify a 5.3-kb PCR product with a primer upstream of the *Lgr3*^*MB06848*^ position (green forward arrow, Supplementary Figs 1a–c and 15a,b) and another on exon 11 (purple arrow, Supplementary Figs 1a–c and 15a,b). Instead of the predicted >5.3 kb product, we obtained a ∼1.6 kb product (Supplementary Figs 1c and 15b), demonstrating that *Lgr3*^*ag1*^ is a large deletion of ∼3.8 kb in the *Lgr3* locus (Supplementary Fig. 1d). This alone indicates that at least 69 aminoacids (aa; I_327_–T_395_) are lacking in the *Lgr3*^*ag1*^ leucine-rich repeat domains, which are critical for relaxin ligand binding in vertebrate relaxin receptors^8^.

Next, we performed reverse transcriptase (RT)–PCR analyses with mRNA isolated from *Lgr3*^*ag1*^ and controls *Lgr3*^*+/+*^ and the original *Lgr3*^*MB06848*^ stock. *Lgr3*^*ag1*^ generated a smear with a major band that was ∼200 bp smaller than the other control genotypes (Supplementary Fig. 2a; Supplementary Fig. 2b,c are RT and *rp49* control reactions, respectively; see also Supplementary Fig. 15c,d). Sanger sequencing determined that the *Lgr3*^*ag1*^ deletion leads to usage of an aberrant splicing acceptor within intron 7 and readthrough directly into exon 9 (Supplementary Fig. 2d). The resulting transcript therefore has an intron-encoded PTC that truncates the Lgr3 protein one amino acid after D_326_ (Fig. 1e). We conclude that *Lgr3*^*ag1*^ encodes a severely truncated protein without the 7TM domains and G protein coupling carboxy terminus (Fig. 1e).

### Generation of *pUASP-Lgr3* and *pUASP-dilp8* flies

A *Drosophila* and *Homo sapiens* codon-optimized complementary DNA (cDNA) corresponding to full-length *Lgr3* was synthetized *de novo* and cloned into *pUASP*^47^ using KpnI and NotI sites to make *pUASP-Lgr3.* This plasmid was injected into *w*^*1118*^ and two independent insertions were tested, *pUASP-Lgr3a (M3, on Chr III)* and *pUASP-Lgr3b (M6, on Chr II).* A similar protocol was used to place the *dilp8::3xFLAG* construct described in reference #3 into *pUASP,* making *pUASP-dilp8a.*

### CRISPR/Cas9 experiments

We used a CRISPR/Cas9-mediated homologous repair strategy^15^,^16^ to tag the endogenous Lgr3 protein at its amino terminus with sfGFP^17^ followed by a flexible linker spacer sequence 5′-GSGSGS-3′ (Fig. 3a and Supplementary Fig. 5). The following guide RNAs (gRNA1 and gRNA2, designed with http://tools.flycrispr.molbio.wisc.edu/targetFinder/)^12^ were synthetized and cloned into pU6-BbsI-chiRNA:

gRNA 1

Fw: 5′-CTTCGGAGCACTCAATTCCCACTC (CGG)-3′

Rv: 5′-AAACGAGTGGGAATTGAGTGCTCC-3′

gRNA 2

Fw: 5′-CTTCGCAAACTCAAGTAGAATATCA (CGG)-3′

Rv: 5′-AAACTGATATTCTACTTGAGTTTGC-3′

The PAM regions (CGG) are located right after the forward primers of both gRNAs 1 and 2, as shown above.

As a repair cassette we designed the following sequence, where each colour represents the following:

Orange: Homology region up to *Lgr3* ATG site

Red: *Lgr3* ATG

Green: sfGFP

Blue: Spacer (GSGSGS)

Violet: Homology region after *Lgr3* ATG site

5′-CACTTAAAACTCTTCTCCGCGAGCTGTGAACATTAGCCAAATGAAGTGACAAGAAATTAACGCAAAAATAAAACAAGAAGACGGAGCGGTATAAGAAATAATAATATAAAAACTCAATGAGTCAGCACCGCATCAGCTCCTGCTGCTGTTGTTCTTCTTATTGCTGTTGTTTGTGGGGGCGTGGCCGGAGTGGGAATTGAGTGCTCCTAATGATGAACTCGGTCAAGGAGCCAGTGCAGCCATGGTGGCCAAGTAATTAGATAAGCGAGCGTGCAAAACAGGAGCAAACCGATAAATCGCCATGCGTAAGGGCGAGGAGTTGTTCACGGGAGTTGTGCCCATATTGGTTGAGCTGGATGGAGATGTGAATGGCCACAAGTTCAGTGTGCGGGGTGAGGGAGAAGGAGACGCAACAAACGGTAAGCTGACACTGAAGTTCATTTGTACTACGGGCAAGCTCCCGGTGCCATGGCCCACATTGGTCACCACCCTGACCTATGGCGTGCAATGCTTCGCCCGATATCCAGATCATATGAAGCAGCATGATTTCTTTAAGTCGGCCATGCCCGAGGGTTACGTACAAGAGCGCACTATTAGCTTTAAGGACGACGGTACGTATAAAACCAGGGCTGAGGTGAAGTTTGAGGGTGATACCCTGGTGAACCGCATTGAATTGAAGGGCATCGATTTTAAGGAGGACGGCAACATCCTGGGCCACAAGCTCGAATATAATTTTAATAGCCATAATGTTTACATTACCGCGGACAAGCAGAAGAATGGAATTAAGGCTAATTTCAAGATCCGACATAATGTGGAGGACGGATCCGTTCAGTTGGCCGATCACTACCAGCAAAACACCCCCATCGGAGATGGCCCCGTCCTGCTGCCCGATAACCACTACCTGAGTACCCAGTCCGTCCTGTCGAAGGATCCTAATGAGAAGCGGGATCATATGGTGCTGCTGGAGTTTGTGACTGCCGCCGGCATAACGCATGGAATGGACGAGCTGTATAAAGGCTCCGGTAGTGGTTCCGTCTACGGCAGGAGCATCGCCGTAGGCTTCTGTCTGATGACCGTCGTCCTTCTGCTGGCCGCCGTGATATTCTACTTGAGTTTGGGTGAGTCCTTAGAGTGATGTCCTTTCAAAATTCCATCATTCGCAAACCTAAATAATTTCTGAATCAAGAATGTTCAAAATCTTAGCAATTATTATACGCATAATTTGTGAAACTACTTAAAGTTCTTTTAAAACTTGAGCTGCTGTAAATTTCTATATATACTTTCGTATCCTTAAAGGGTTCCTTCGCTTGAAGCAAAAACCAAAATCAAATTCCAAACTGCAAA-3′

The repair cassette was synthetized *de novo* into a pUC57 plasmid and co-injected with the two gRNAs into the stock y[1] M{w[+mC]=Act5C-Cas9.P}ZH-2A w[*], which strongly and ubiquitously expresses a human codon-optimized cas9 (ref. 15). The adults originating from this injection were separately crossed to a *MKRS/TM6B* stock. Males from each F1 cross were separately crossed again to MKRS/TM6B. When larvae appeared in the vials, we extracted gDNA from the F1 male progenitor and made PCR reactions using the primer pairs:

Lgr3_crispr_testF: 5′-CCAATAACTTTAAGCCGTCTGTG-3′

SuperfolderGFP_R: 5′-CAGCACCATATGATCCCGCT-3′

SuperfolderGFP_F: 5′-CCTATGGCGTGCAATGCTTC-3′

Lgr3_crispr_testR: 5′-TATAGCTGTGCGAATTTCTCGAT-3′

These primers were designed to indicate the correct insertion of the sfGFP repair cassette from both sides in the genome. Positive hits were sequenced (*ag5-ag9*). Ten negative hits were also sequenced and two were retained as background controls (*ag10* and *ag11*). The *y*^*1*^ M{w[+mC]=Act5C-Cas9.P}ZH-2A w* cassette was removed by selecting against eye colour and stocks were kept as homozygous stocks, except for allele *ag8,* which was kept balanced over TM6B.

### Measurement of the developmental timing of pupariation

Male and virgin female flies aged 3–10-day-old were crossed and 1–2 days later transferred to an agar plate with yeast–sucrose paste (1:1). The next morning, the flies were transferred to a fresh plate to lay eggs for 3–6 h, depending on the experiment. To control for overcrowding, between 10 and 30 larvae were transferred to vials containing normal *Drosophila* food in 3–12 batches depending on the experiment. Survey of pupae consists in counting the number of pupae in each time interval. The final total *n* of pupae for each genotype in each experiment is depicted in the respective figures. For pupariation time assays, we aimed at obtaining at least 30 individual larvae per genotype, yet some genotypes were sick or difficult to obtain due to balancer chromosomes, yielding less larvae of the correct genotype to score. Male and females were scored together. The time of pupariation of each larvae was determined and genotypes were compared with the Kruskal–Wallis non-parametric test followed by *post hoc* ranks test after Conover, with *α*=0.01 or 0.05, as indicated in each figure, using the software Infostat. Data were plotted in box plots representing median, 25 and 75% quartiles, and 5–95% percentiles as whiskers. Data points falling outside of the 5–95% interval were plotted as outliers. The Kruskal–Wallis test is a rank-based nonparametric test that can be used to determine statistically significant differences between two or more groups. It is a nonparametric alternative to the one-way analysis of variance, and as such, normal distribution is not required. Developmental timing assays were performed at 25 °C under constant light, except for experiments reported in Fig. 2a and Supplementary Fig. 3b, which were done in a 12-h light/dark cycle, and EMS experiments (see below), which were done in the dark to minimize the reaction of EMS with light.

### Duration of the third instar

Egg collections were performed on normal food plates and larvae were reared at controlled densities without additional yeast (about 200 eggs per 60 mm diameter normal fly medium plate). Newly molted third instar larvae were collected every 2 h as described previously^48^,^49^. After staging, collected larvae were raised in a normal cornmeal/molasses medium at 20–30 larvae per vial without additional yeast. Pupariation time was observed every 2 h until all treated larvae pupariated or died. Male and females were scored together. We defined pupariation as cessation of movement with evaginated spiracles. These experiments were performed at 25 °C under constant light.

### EMS treatment

Larvae were collected as described above and transferred 72 h after egg laying to fresh food with 10 mM of EMS (Sigma) or PBS as control. Developmental time was measured as indicated above. EMS food was prepared as follows: food was melted and cooled to 55 °C, an appropriate volume of freshly made EMS stock solution in PBS was added and thoroughly mixed and 3 ml per tube were dispensed. EMS and PBS tubes were kept in the dark as much as possible throughout the experiments.

### Immunofluorescence staining

Brains of wandering third instar larvae or first instar larvae were dissected in cold PBS, fixed for 30 min in 4% paraformaldehyde, rinsed with PBS with Triton (0.3%) (PBST), incubated with primary antibody overnight and with fluorescently labelled secondary antibody for 2–24 h in PBST with 1% bovine serum albumin. Nuclei were counterstained with DAPI (Sigma) and tissues were mounted in Fluoromount-G (Southern Biotech). Antibodies used were: mouse anti-GFP 1:200 (DSHB, 12E6), mouse anti-nc82 1:250 (DSHB), mouse anti-HA 1:50 (Santa Cruz, 12CA5), rabbit anti-GFP 1:200 (Life technologies, A11122), rabbit anti-Dilp7 1:5000 (gift from I. Miguel-Aliaga^21^) and rat anti-Dilp5 1:400 (gift from P. Leopold^50^). Images were obtained with a Zeiss LSM 710 Confocal Microscope and images were analysed using FIJI software^51^. Typically 5–10 brains were mounted for observation and 1 representative image per genotype is depicted in figures. Brains from male and female larvae were scored together.

### Adult wing measurements

Pairs of the left and right wings of male individuals reared at 29 °C and rinsed with ethanol were dissected in a glycerol/ethanol solution and mounted in glycerol. Photos were obtained in a Zeiss Axiovert 40 CFL microscope. The wing areas and wing lengths were calculated as previously described^3^,^52^, using Fiji. We used the FAi to assess intra individual size variations between the left and right wings^52^. Namely, FAi=Var(Ai), where Ai is the normalized difference between left and right wing areas of each individual Ai=*A*_left_-*A*_right_/[(*A*_left+_*A*_right_)/2]. Results were compared statistically using the F-test for the significance of the difference between the FAi of the samples, an appropriate test for dispersion^3^,^52^. Bonferroni corrections (*α*/*n*) for multiple comparisons was applied to Figs 5d,f, using *α*=0.05.

To control for measurement errors, we measured the area of the same wing three times. Values obtained were 18310.2±30.5 a.u., which gives a coefficient of variation (CV) of 0.17%, which is smaller than the CV of wing areas of control *Lgr3*^*+/+*^ by a factor of ∼23 (CV=3.88%, *n*=48, flies reared at 29 °C).

### General molecular biology

gDNA was extracted from a group of flies or single flies^53^. Briefly, the flies were macerated using pellet pestles and homogenized in 100 μl DNA extraction buffer (1 M Tris-HCl at pH 8.2, 0.5 M EDTA, 5 M NaCl). Then, we added 1 μl protease K 50 ng μl^−1^ (Roche), and incubated the mixture at 37 °C for 1 h, followed by 95 °C for 5 min, to inactivate the protease.

RNA was extracted using the Direct-zol RNA MiniPrep kit (Zymo Research), following manufacturer's instructions. The material used for the RT–PCR experiments described in Supplementary Fig. 2 was obtained from 15 virgin males aged between 3–7 days, and was macerated using pellet pestles and homogenized in 500 μl TRI Reagent and centrifuged at 12,000*g* for 1 min, to lower tissue debris. An extra DNAse treatment (Turbo DNA-free kit, Ambion, Life Technologies) was performed to reduce gDNA contamination. cDNA synthesis was performed using the Maxima First Strand cDNA Synthesis Kit for RT–quantitative PCR (Thermo Scientific), following manufacturer's instructions.

For this study, PCR and RT–PCR primers were designed and their specificity tested using Primer BLAST or Primer3. A T100 Thermal Cycler (Bio-Rad) was used for performing the PCR steps. The following primers were used for PCR analyses described in Supplementary Figs 1 and 2.

Lgr3_salto_fw (expected product size 866 bp)

Fw: 5′-CCGACGCCTTGCTGCTAACT-3′

Rv: 5′-TTTATGGAGCGGGCGTGGTC-3′

Lgr3_exonshort Lgr3 (expected product size 331 bp)

Fw: 5′-CCGACGCCTTGCTGCTAACT-3′

Rv: 5′-GTGCGTTATGAGGTTGTGCTG-3′

Lgr3_exon3p Lgr3 (expected product size 240 bp)

Fw: 5′-CGCCTTGTCGGTAATCCCAT-3′

Rv: 5′-GTGGCTCCATTAAACTGCTGC-3′

Lgr3_exons (expected product size 5,307 bp)

Fw: 5′-CCGACGCCTTGCTGCTAACT-3′

Rv: 5′-CAAAGACCACCAACCAGGCGTA-3′

*rp49* (control)

Fw: 5′-TTGAGAACGCAGGCGACCGT-3′

Rv: 5′-CGTCTCCTCCAAGAAGCGCAAG-3′

qRT–PCR experiments were performed using Lightcycler 96 (Roche) using the FastStart Essential DNA Green Master dye and polymerase (Roche). The final volume for each reaction was 10 μl, consisting of 5 μl of dye and polymerase (master mix), 2 μl of 10 × diluted cDNA sample and 3 μl of the specific primer pairs. The following primers were used:

*rp49* (control)

Fw: 5′-TTGAGAACGCAGGCGACCGT-3′

Rv: 5′-CGTCTCCTCCAAGAAGCGCAAG-3′

Lgr3

Fw: 5′-GCTGGGTGCCCATCATCGTTAT-3′

Rv: 5′-CAAAGACCACCAACCAGGCGTA-3′

InR

Fw: 5′-TGTCAGCTGCACAATAATAGGC-3′

Rv: 5′-TGCACTTTTCAGGGCATTT-3′

Drl

Fw: 5′-CGGAGTTCCATACCCAGATTAC-3′

Rv: 5′-GCCTCTTGTTATATTTACAGGTCTTGG-3′

Primer efficiency for qRT–PCR was tested by serial dilution. Data were expressed as %*rp49* according to the formula: %*rp49*=(2̂−(ΔCqLgr3−ΔCqrp49)) × 100. The geometric mean±s.e.m. of three biological repeats was used and data were analysed by one-tailed unpaired Student's *t*-test using Bonferroni corrections (*α*/*n*) for multiple comparisons, using *α*=0.05. This test assumes the independent samples have equal variances.

### Ligand–receptor capture

LRC technology allows the retrieval of previously unknown receptors for different soluble peptides and proteins in native cellular contexts and was performed essentially as previously described^27^,^28^, using a kit ‘CaptiRec' from Dualsystems Biotech AG. The LRC technique relies on a tri-functional and biocompatible chemoproteomic reagent (named TriCEPS)^27^ that allows coupling to the peptide ligand-of-interest, direct covalent capture of gently oxidized glycosylated receptors on living cells and a biotin tag for purification^27^. Briefly, TriCEPS reagent was coupled with 100 μg of mature Dilp8 peptide (Phoenix Pharmaceuticals, Inc)^29^ or with human insulin or glycine, as positive and negative controls, respectively, following manufacturer's instructions. TriCEPS–ligand complexes were assayed in triplicates at 100 μg per 10^9^ *Drosophila* SL2/DL2 cells (ATCC) cultured in Schneider medium supplemented with 10% foetal bovine serum. The cells were gently oxidized with NaIO_4_ and TriCEPS–ligand complexes were incubated with the cells for 1.5 h to allow capture of oxidized glycomoieties. Following cell lysis, trypsinization and biotin-mediated affinity purification of ligand–receptor peptides, the glycosylated peptides were selectively released using PNGase F, and samples were analysed by Dualsystems Biotech AG on an LTQ Orbitrap XL (Thermo Scientific) spectrometer fitted with an electrospray ion source. The samples were measured in data-dependent acquisition mode in a 40-min gradient using a 10-cm C18 column. Peptide identifications were filtered to a false-discovery rate (FDR) of ≤1% (Comet MS/MS search engine, Release 25 September 2014 (ref. 54) embedded in the TransProteomicPipeline (TPP v4 POLAR VORTEX rev 0, Build 201402281256) (ref. 55)) and quantified using an MS1-based label-free approach (Progenesis QI for proteomics; nonlinear Dynamics). A total of 114 glycopeptides could be identified and quantified in our experiments, representing 87 glycoproteins (Supplementary Tables 1 and 2). For statistical analyses, the six individual samples per data set were analysed with analysis of variance with *P* values adjusted for multiple comparisons to control the experiment-wide FDR (MSstats v2) (ref. 56). The adjusted *P* value obtained for every protein is plotted against the magnitude of the fold enrichment between the two experimental conditions. The area in the volcano plot that is limited by an enrichment factor of fourfold or greater and an FDR-adjusted *P<*0.05 is defined as the receptor candidate space.

### General study design and statistics

In all experiments reported in this work, no data point was excluded. All data points, including outliers, are represented in the figures and were used in the statistical analyses. No blinding was done and no particular randomization method was used to attribute individuals to experimental groups.

## Additional information

**Accession codes:** DNA sequence data has been deposited in GenBank under accession codes KT321103-KT321113.

**How to cite this article:** Garelli, A. *et al.* Dilp8 requires the neuronal relaxin receptor Lgr3 to couple growth to developmental timing. *Nat. Commun.* 6:8732 doi: 10.1038/ncomms9732 (2015).

## Supplementary Material

Supplementary Information Supplementary Figures 1-15 and Supplementary Tables 1-2
