# Supplementary material for: Dilp8 requires the neuronal relaxin receptor Lgr3 to couple growth to developmental timing
Source: Nat Commun. 2015 Oct 29;6:8732. doi: 10.1038/ncomms9732 (PMC4640092; doi:10.1038/ncomms9732)
Supplement: Supplementary Information — Supplementary Figures 1-15 and Supplementary Tables 1-2 [file ncomms9732-s1.pdf]

Supplementary Figure 1

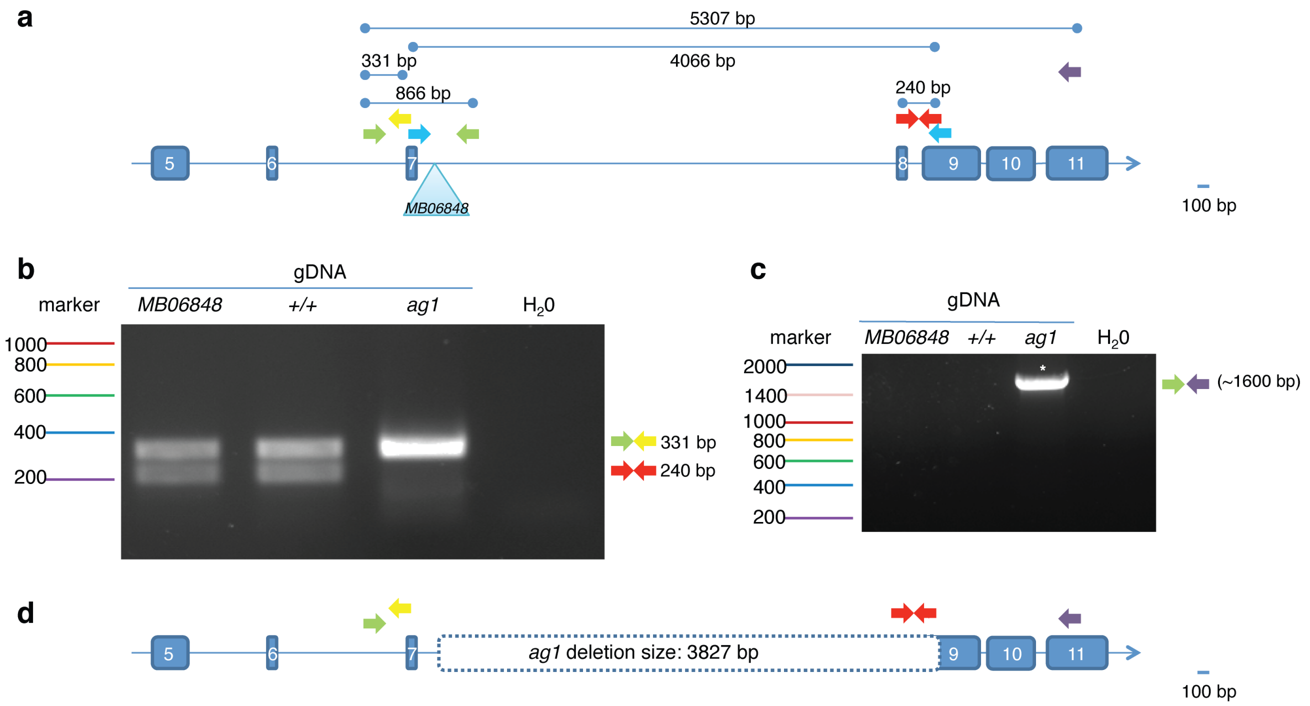

Supplementary Figure 1: Molecular characterization of the *Lgr3*^{MB06848} remobilization lines. a, Scheme with key sets of primers used, as well as the insertion site. In this scheme, the *Lgr3* locus transcribes from left to right. Coding exons are numbered. Colored arrows are shown the approximate location of the primers used. The blue set of primers was used to identify the deletion breakpoints. **b,** Electrophoretic pattern of PCR amplification products of the precise and imprecise excision *Lgr3*$^{+/+}$ and *Lgr3*^{ag1}, respectively, which shows that the deletion occurred downstream (to the right) of the insertion site. **c,** Electrophoretic band of PCR amplification product of *Lgr3*^{ag1}, from which we later obtained the gDNA sequence and identified a deletion of approximately 3.8 kb. **d**, Scheme of the *Lgr3*^{ag1} deletion. The deletion removes most of the intron and completely removes exon 8 and partially exon 9. The negative control used in the different experiments was dH_2O, showing no PCR amplifications, as expected. Molecular markers, bp. Images of the full gels with molecular markers for panel **b** and **c** are shown in Supplementary Fig. 15.

Supplementary Figure 2

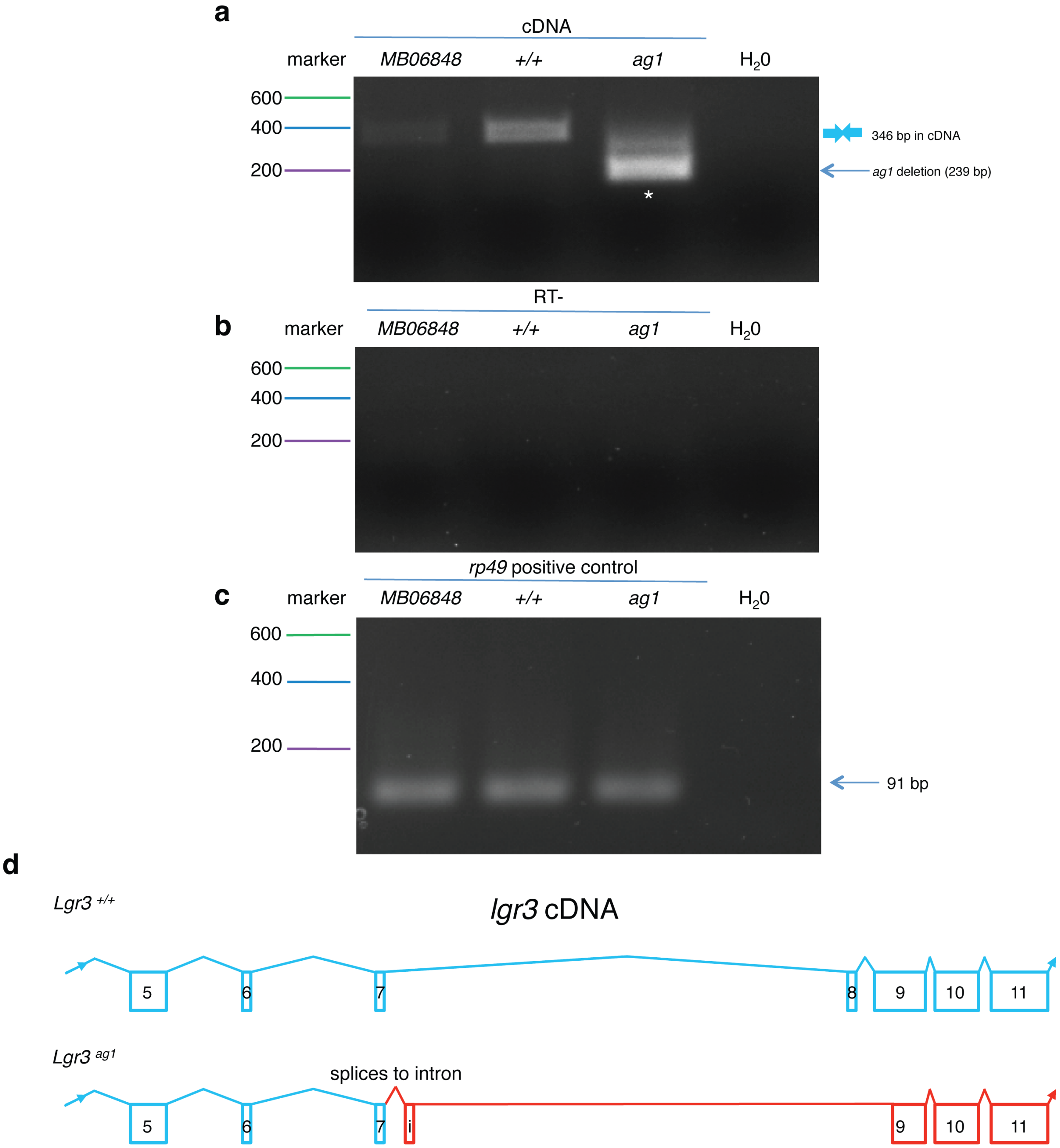

Supplementary Figure 2: RT-PCR amplification pattern of Lgr3 mutations and controls using mRNA isolated from males. a, Electrophoretic pattern of PCR amplification products of *Lgr3*$^{+/+}$ and *Lgr3*^{ag1}, with the set of primers *Lgr3_around* (set of primers in blue in Supplementary Fig. 1). The *Lgr3*^{ag1} mutant gave a smear and a major product at around 200 bp, consistent with a non-spliced transcript reading at least partially directly through the deletion breakpoint (which was confirmed by Sanger sequencing, see below). **b**, RT negative control, showing no amplification products, ensuring that any gDNA contamination, if present, was below detection levels. **c**, The housekeeping gene *rp49* was used as a positive control for the RT-PCR. The negative control used was dH_2O. **d**, Schematic representation of the *Lgr3* cDNA in the *Lgr3*^{ag1} mutant obtained by Sanger sequencing and in the *Lgr3*$^{+/+}$ control line. Blue, normal splicing and exon reading frames. Red, aberrant splicing and frame shifted exons. Images of the full gels with molecular markers for panels **a**, **b** and **c** are shown in Supplementary Fig. 15.

Supplementary Figure 3

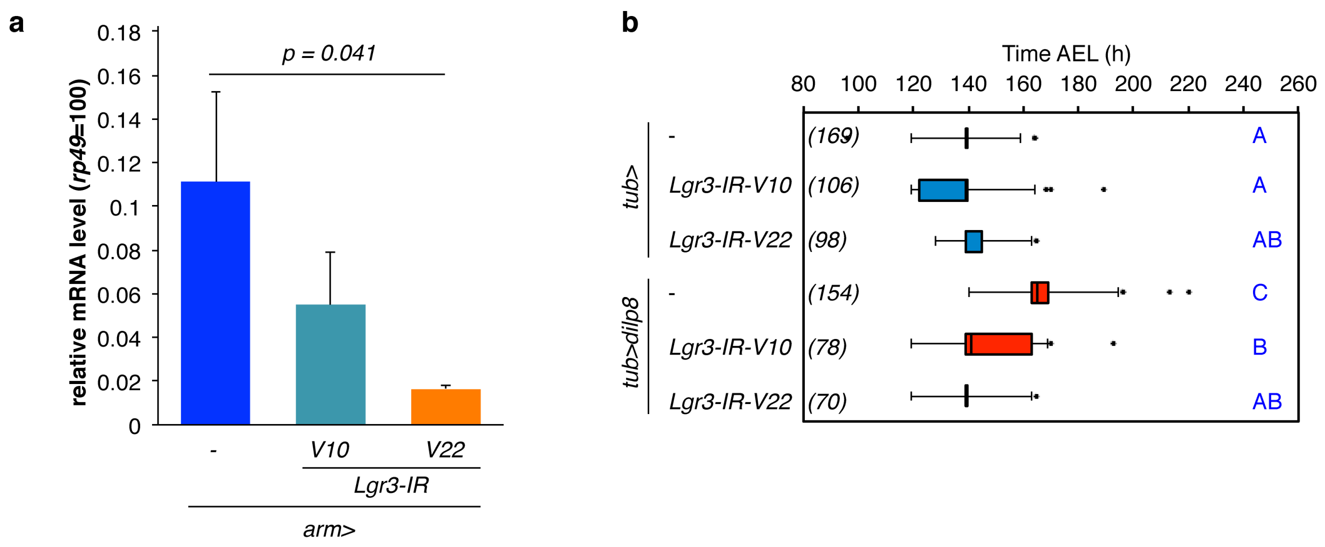

Supplementary Figure 3: *Lgr3* mRNA quantification by qRT-PCR following RNAi against *Lgr3* and suppression of the Dilp8-dependent delay by RNAi against *Lgr3*. a, Two different lines (*Lgr3-IR-V10*^{JF03217} and *V22*^{GL01056}) were expressed under the control of the ubiquitous Gal4 driver, *arm>*. mRNA was isolated from adult males. Values represent the geometric mean +/- standard errors of the mean (n=3 biological repeats) of *Lgr3* mRNA levels relative to *rp49* levels (*rp49* = 100). *Lgr3* transcript levels are between 3-4 orders of magnitude less abundant than *rp49*. While both RNAi lines reduce the steady state *Lgr3* mRNA levels relative to the control line, only the *V22* RNAi leads to a statistically significant result (unpaired one-tail Student's t-test; t = 1.19, df = 4 for arm> x arm>Lgr3-IR-V10; and t = 2.31, df = 4 for arm> x arm>Lgr3-IR-V22). p = 0.1499 for the *V10* RNAi. **b,** Two different RNAi lines against *Lgr3*, suppress the delay caused by expression of Dilp8 under the control of the ubiquitous *tub>* driver. Boxplots (see Methods) showing pupariation time of (*N*) larvae obtained from 3 egg layings. Whiskers are 5 and 95% percentiles. Dots, outliers. p < 0.0001, Kruskal Wallis test. Genotypes sharing the same letter (blue) are not statistically different at alpha = 0.01, Conover post-hoc-test. Degrees of freedom, H and C values for the Kruskal Wallis test are df = 5, H = 192.3, C = 0.93.

Supplementary Figure 4

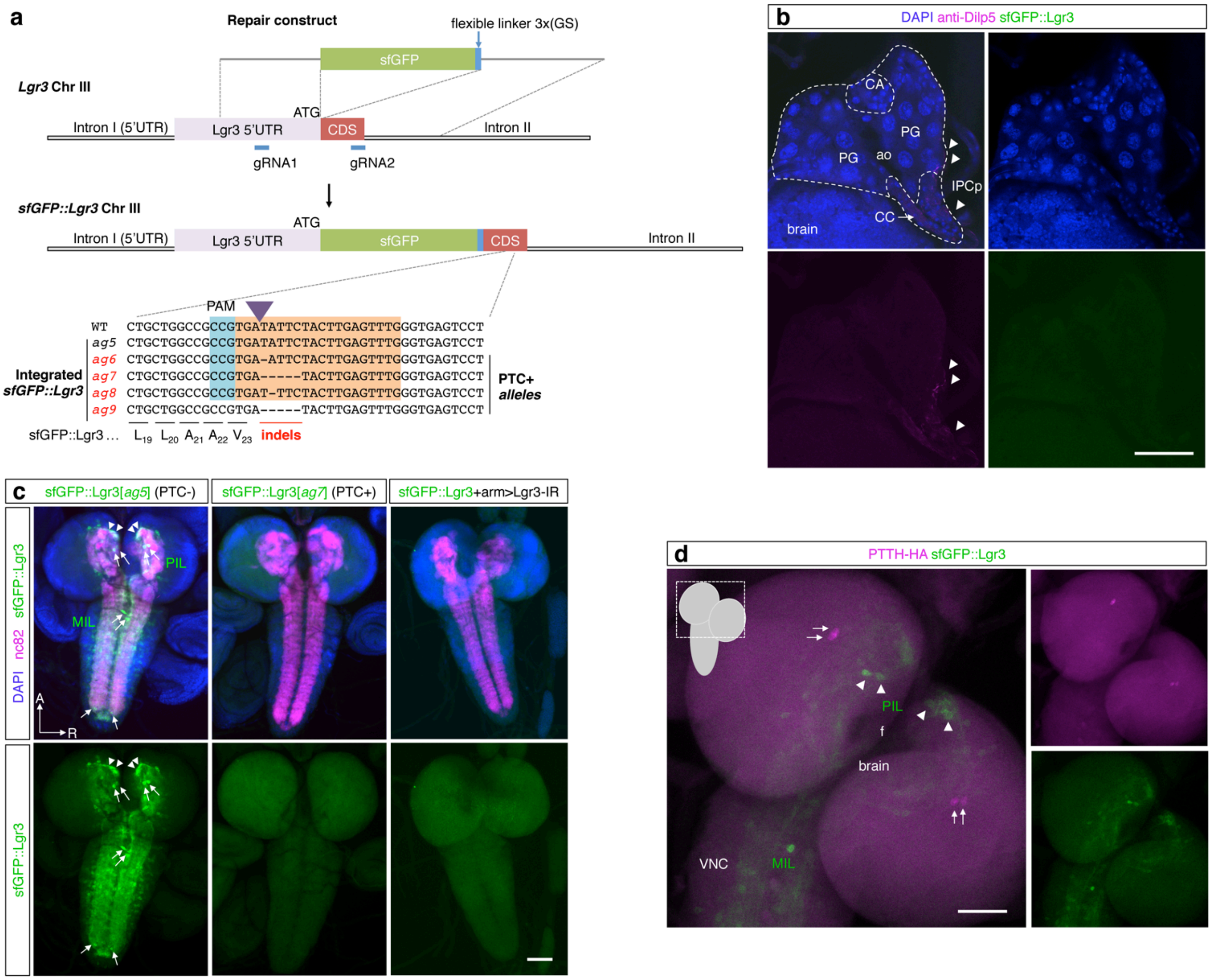

Supplementary Fig. 4: Strategy used to generate the sfGFP knock-in using CRISPR/Cas9-mediated repair. **a**, The localization of the two guide RNAs (gRNA1 and 2) used are depicted. One integrated allele, *ag5* (*sfGFP::Lgr3*), did not contain indels in the coding sequence (CDS) of *Lgr3* exon 2 downstream of the sfGFP sequence. The other alleles contained the depicted PTC-inducing indels in the *Lgr3* exon 2. Some alleles also contained between 1-6-nucleotide deletions in the non-coding region upstream of the *Lgr3* gene targeted by gRNA1, except for *ag8,* which contained a much larger 350-bp deletion, which also removed the ATG and part of the sfGFP insert. *ag7* and *ag9* are independent alleles missing 5 and 1 nucleotides in the upstream gRNA1 region. The WT sequence corresponds to allele *ag10* (see Methods). **b**, Single confocal section of the ring gland of an *sfGFP::Lgr3* animal stained for anti-Dilp5 (magenta) and anti-GFP for sfGFP::Lgr3 (green). Blue, DAPI counterstain. The three endocrine glands of the ring gland are depicted: CA, corpora allata, PG, prothoracic gland, CC, corpora cardiaca. ao, aorta foramen. Anti-Dilp5 stains the CC and the ring gland projections of the IPC (IPCp, arrowheads). **c,** Sum of confocal z-stack slices of the CNS of a 3rd instar larva stained with anti-GFP (green) to show sfGFP::Lgr3 expression (green) and with anti-nc82 (magenta) and DAPI (blue) counterstains to show the synapses (neuropil) and nuclei, respectively. The panel of the right is reproduced here from Fig. 3c (Main text), to ease direct comparison with the right panels. Arrows point to two bilateral pairs of PIL neurons (top), to the MIL neurons in the midline of the VNC (middle), and the distal VNC pair (bottom). Arrowheads point to the proximal projections of the PIL neurons. sfGFP::Lgr3 is also expressed in ~170 other cell bodies, but at a lower level than in PIL and MIL neurons. No anti-GFP staining is detectable in the CNS of an animal carrying an

sfGFP::Lgr3 insertion with a PTC+ indel [*ag7*] (middle panel) or in animals expressing RNAi against *Lgr3* (right panel). **d,** Sum of confocal z-stack slices of the CNS of an animal expressing *sfGFP::Lgr3* (stained with anti-GFP, green) and an HA-tagged PTTH, detected with an antibody against HA (magenta). PTTH is expressed in two bilateral neurons (arrows) in the brain, which do not show detectable sfGFP::Lgr3 expression. PIL neurons are depicted by arrowheads. f, esophageal foramen. Scale bars = 50 μm.

Supplementary Figure 5

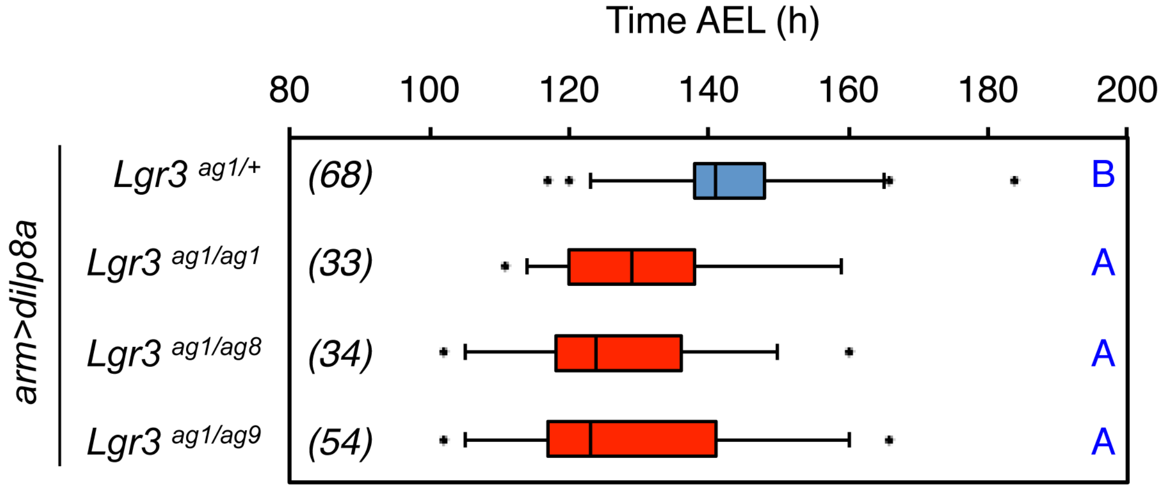

Supplementary Figure 5: PTC+ *Lgr3* mutations (*ag8* and *ag9*) generated by the CRISPR-Cas9 strategy suppress the delay induced by ectopic Dilp8 expression (using *arm>dilp8a*). Boxplots (see Methods) showing pupariation time of (*N*) larvae obtained from 5 egg layings. Whiskers are 5 and 95% percentiles. Dots, outliers. $p < 0.0001$, Kruskal Wallis test. Genotypes sharing the same letter (blue) are not statistically different at alpha = 0.01, Conover post-hoc-test. Degrees of freedom, H and C values for the Kruskal Wallis test are df=3, H=40.17, C=0.99. $Lgr3^{ag/+}$ and $Lgr3^{ag1/ag1}$ are wild-type and null controls for *Lgr3* activity, respectively.

Supplementary Figure 6

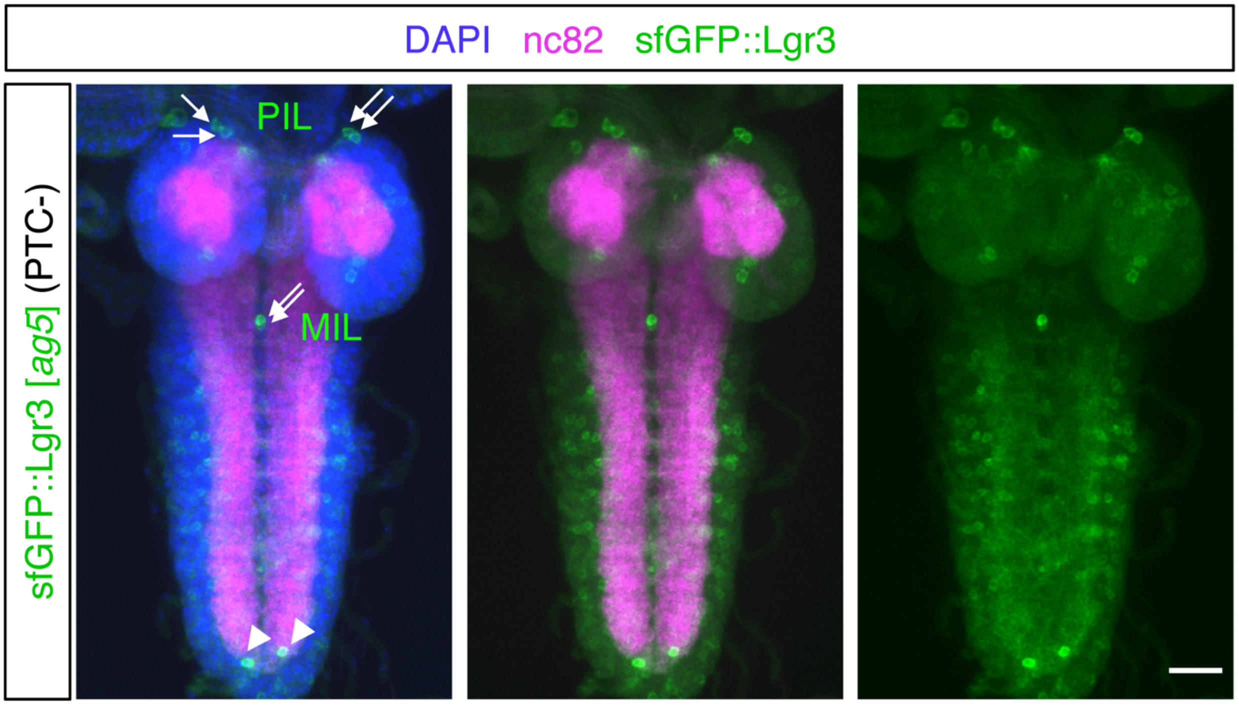

Supplementary Figure 6: *sfGFP::Lgr3* is expressed in L1 stage larvae. Sum of confocal z-stack slices of the CNS of a 1st instar larva (L1) stained with anti-GFP (green) to show sfGFP::Lgr3 expression (green) and with anti-nc82 (magenta) and DAPI (blue) counterstains to show the synapses (neuropil) and nuclei, respectively. MIL and PIL neurons are present at anatomically defined sites (arrows). An additional pair of neurons expressing high levels of sfGFP::Lgr3 is notable in the VNC of L1. Scale bar = 20 µm.

Supplementary Figure 7

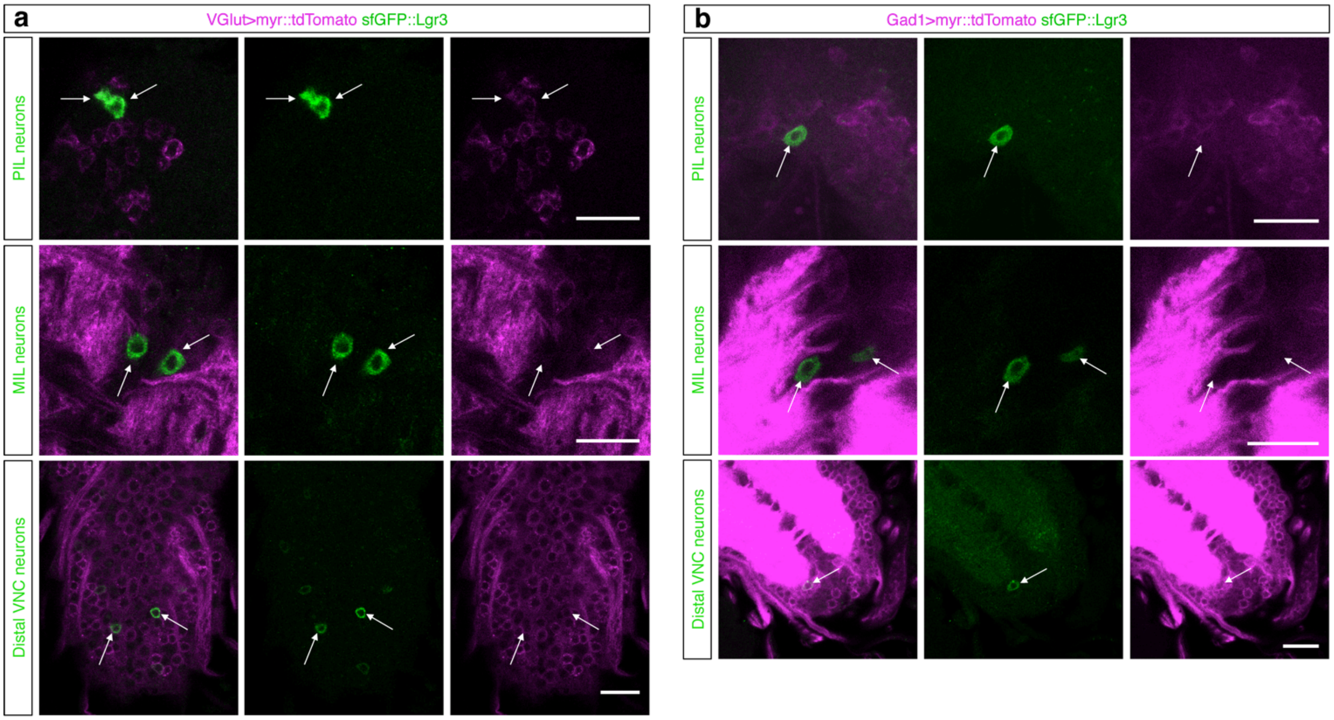

Supplementary Figure 7: Overlap between *VGlut>myr::tdTomato* and *Gad1>myr::tdTomato* and *sfGFP::Lgr3* expression patterns. The neurons expressing highest levels of *Lgr3* (PIL, MIL and distal pair of VNC neurons, arrows in upper, middle and lower panels, respectively, in **a** and **b**) are labeled with sfGFP::Lgr3 (anti-GFP, green) and *VGlut>* and *Gad1>* neurons were visualized with a *UAS-myr::tdTomato* reporter (magenta). Neither *VGlut>* or *Gad>* expression were detected in MIL neurons. However in PIL and the Distal VNC neurons, some faint expression could not be ruled out. Notice that the red channels (magenta) were overexposed in some panels to verify fainter expression. Scale bars = 20 μm.

Supplementary Figure 8

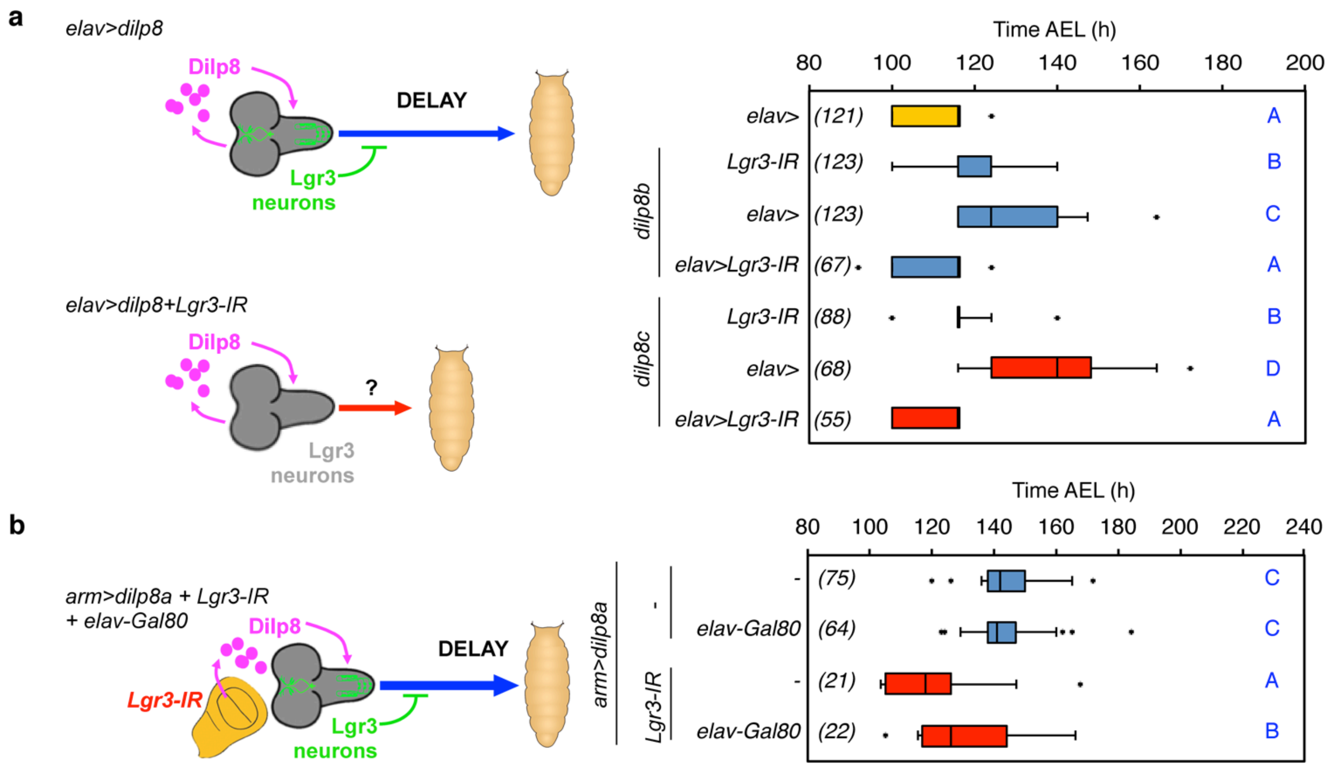

Supplementary Figure 8: *Lgr3* is required in neurons to convey the Dilp8-dependent developmental delay. a, Neuronal expression of *dilp8* using two different transgenes (*dilp8b* or *dilp8c*) causes a delay that is suppressed by neuronal RNAi against *Lgr3*. **b**, The rescue of the developmental delay caused by ectopic expression of Dilp8 using *arm>dilp8a* in the presence of RNAi against *Lgr3* can be partially impeded by inhibiting Gal4 in neurons using *elav-Gal80* transgene. **a,b,** Boxplots (see Methods) showing pupariation time of (*N*) larvae obtained from 6 and 3 egg layings for panels **a** and **b,** respectively. Whiskers are 5 and 95% percentiles. Dots, outliers. $p < 0.0001$, Kruskal Wallis test for all panels. Genotypes sharing the same letter (blue) are not statistically different at alpha = 0.01 and 0.05 for **a** and **b**, respectively, Conover post-hoc-test. Degrees of freedom, H and C values for the Kruskal Wallis tests are df = 6, H = 237.4, C = 0.82; df = 3, H = 40.89, C = 0.99, for panels **a** and **b,** respectively.

Supplementary Figure 9

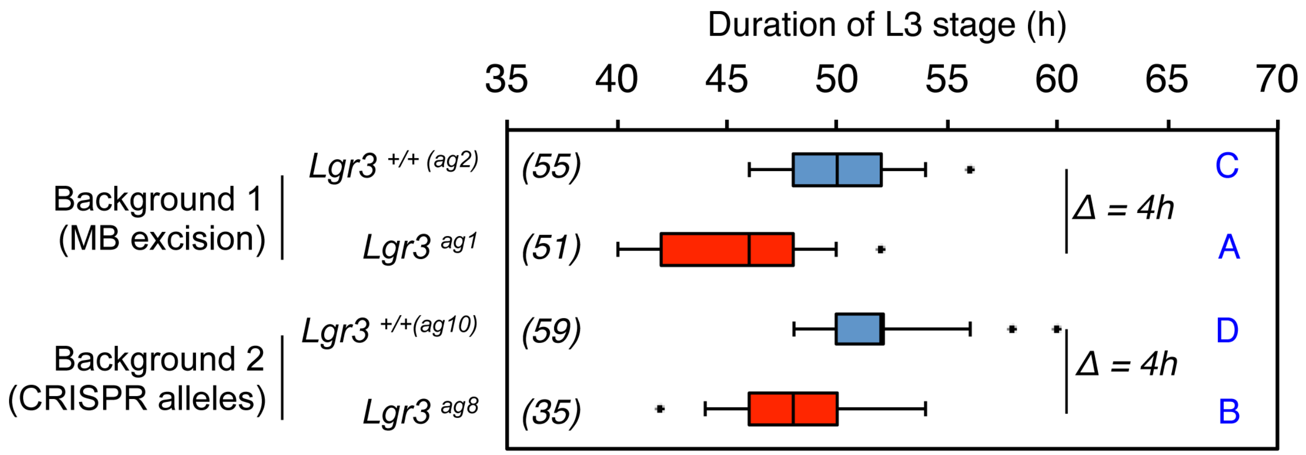

Supplementary Figure 9: Duration of L3 stage of Lgr3 null mutations in two different backgrounds. Boxplots (see Methods) showing pupariation time of (*N*) resynchronized larvae obtained from 3 egg layings. Whiskers are 5 and 95% percentiles. Dots, outliers. $p < 0.0001$, Kruskal Wallis test. Genotypes sharing the same letter (blue) are not statistically different at alpha = 0.01, Conover post-hoc-test. Degrees of freedom, H and C values for the Kruskal Wallis test are df = 3, H = 99.91, C=0.97.

Supplementary Figure 10

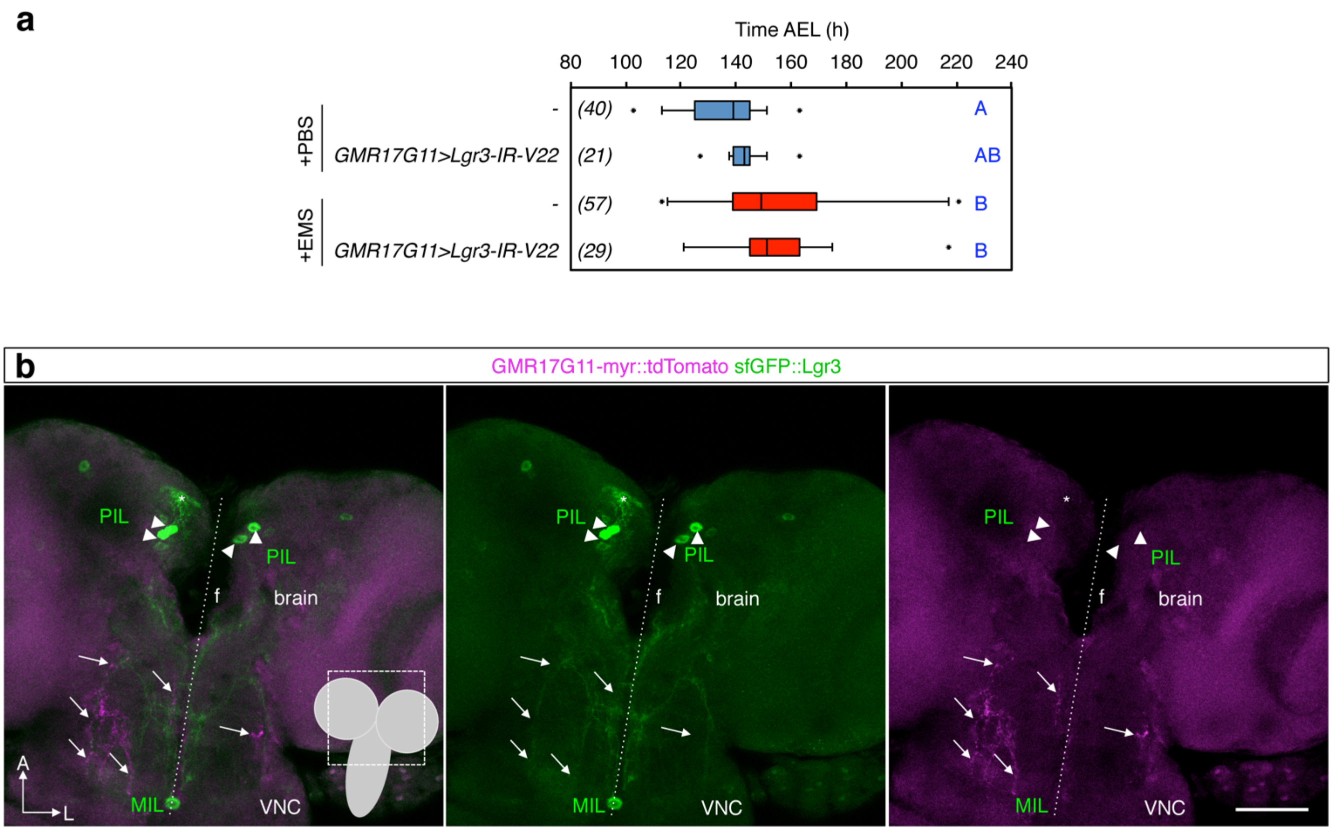

Supplementary Figure 10: The Janelia Gal4 driver *GMR17G11* does not rescue the EMS-dependent delay when driving *Lgr3-IR* and is not expressed in the PIL or MIL neurons. a, Boxplots (see Methods) showing pupariation time of (*N*) larvae obtained from 6 egg layings. Whiskers are 5 and 95% percentiles. Dots, outliers. $p < 0.0001$, Kruskal Wallis test. Genotypes sharing the same letter (blue) are not statistically different at alpha = 0.01, Conover post-hoc-test. Degrees of freedom, H and C values for the Kruskal Wallis test are df = 3, H = 23.89, C = 0.99. The controls (-) of this panel are the same as reported in Fig. 4a, as both experiments were run in parallel. **b,** Overlap between *GMR17G11>myr::tdTomato* and *sfGFP::Lgr3* expression patterns. Sum of confocal z-stack slices showing Lgr3 positive neurons labeled with sfGFP::Lgr3 (anti-GFP, green). The *GMR17G11>* neurons were visualized with a UAS-myr::tdTomato reporter (magenta). *GMR17G11>* does not drive detectable expression in PIL (arrowheads) or MIL neurons. Arrows depict some of the anteriormost neurites from *GMR17G11>* neurons, which are enriched in the VNC. The midline is depicted with a dotted line. An asterisk depicts the proximal ramifications of the PIL neurons. f, esophageal foramen. Scale bar = 50 μm.

Supplementary Figure 11

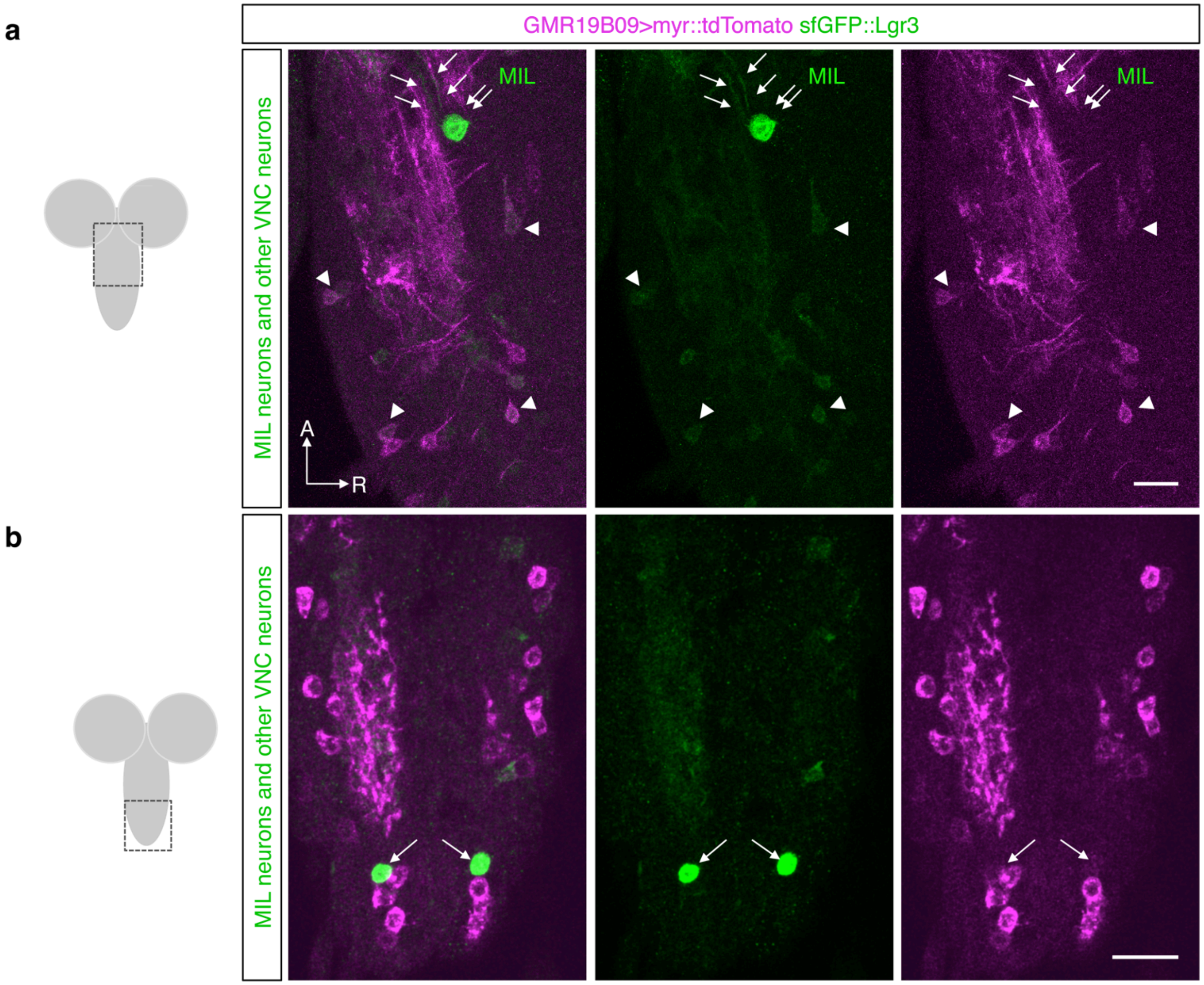

Supplementary Figure 11: Overlap between *GMR19B09>myr::tdTomato* and *sfGFP::Lgr3* expression patterns. MIL neurons and distal pair of VNC neurons (arrows in upper and lower panels, respectively) labeled with sfGFP::Lgr3 (green). The *GMR19B09>* neurons were visualized with a UAS-myr::tdTomato reporter (magenta). **a,** *GMR19B09>* does not drive detectable expression in MIL neurons (magenta) and **b,** only very faint expression in the distal VNC neuronal pair. Scale bars = 20 μm.

Supplementary Figure 12

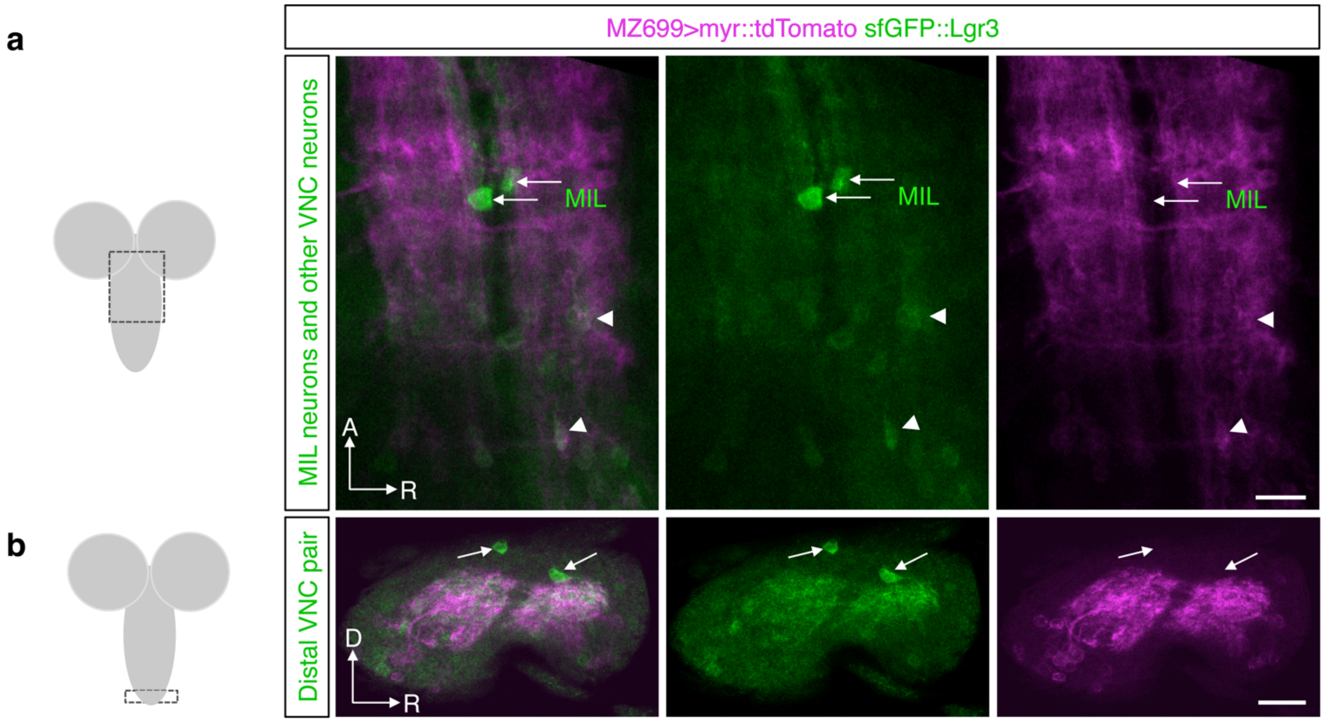

Supplementary Figure 12: Overlap between *MZ699>myr::tdTomato* and *sfGFP::Lgr3* expression patterns. MIL neurons and distal pair of VNC neurons (arrows in upper and lower panels, respectively) labeled with sfGFP::Lgr3 (green) and *MZ699>* neurons were visualized with a *UAS-myr::tdTomato* reporter (magenta). **a,** *MZ699>* does not drive detectable myr::tdTomato (magenta) in MIL neurons (green) or in **b,** the distal VNC neuronal pair (green). Scale bars = 20 µm.

Supplementary Figure 13

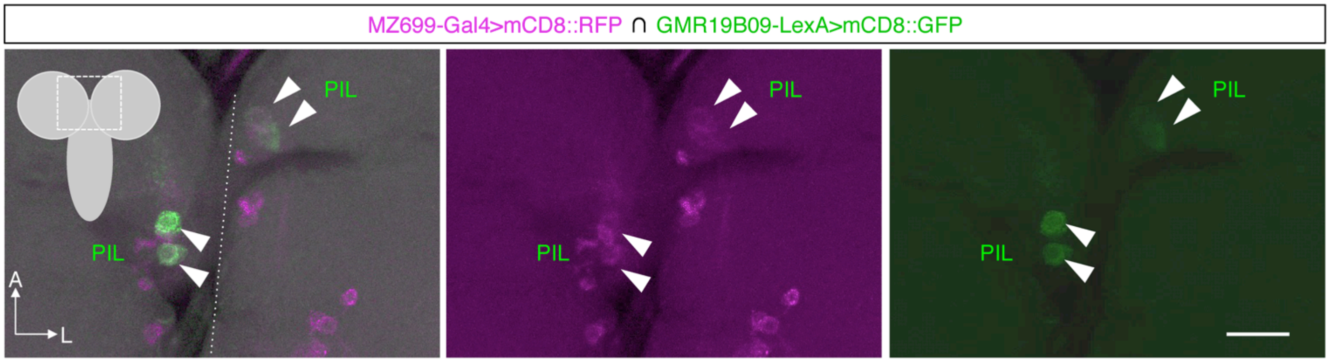

Supplementary Figure 13: Detail of the pars intercerebralis region depicting PIL neurons co-expressing *GMR19B09-LexA* and *MZ699>*. Max-intensity projections of confocal z-stack sections showing the intersectional pattern of *GMR19B09-LexA* driving *lexAop-mCD8::GFP* (green) and *MZ699>mCD8::RFP* (magenta). PIL neurons expressing both drivers are depicted with arrowheads. This is an inset of the image depicted in Fig. 6g, presented with the split channels. Scale bar = 20 µm.

Supplementary Figure 14

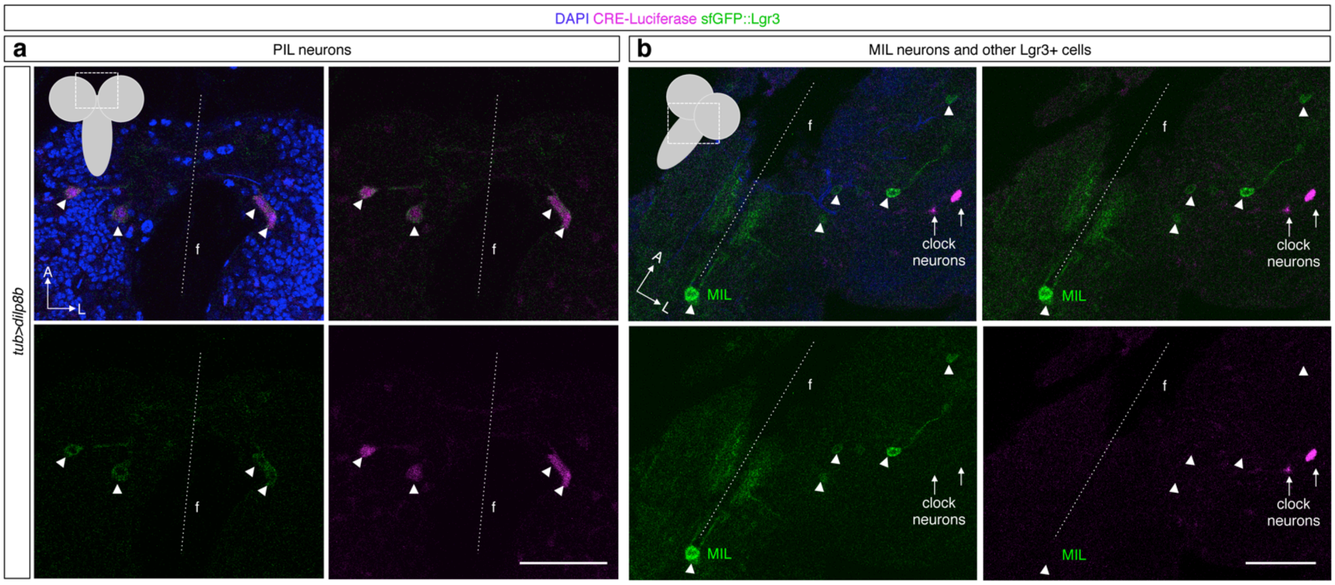

Supplementary Figure 14: Overlap between CRE-luciferase expression and *sfGFP::Lgr3* expression patterns. a, Single confocal slice showing the pars intercerebralis region of a CNS from a larva expressing *tub>dilp8b* and both CRE-luciferase and *sfGFP::Lgr3* reporter transgenes. PIL neuron cell bodies (arrowheads) express both CRE-luciferase (anti-luciferase, magenta) and sfGFP::Lgr3 (anti-GFP, green). Background anti-luciferase staining in glial cells is also detectable throughout the CNS. f, esophageal foramen. Midline is labeled with a dashed line. This image is an inset of the same brain depicted in Fig. 7a, copied here to allow direct comparison with panel b. **b**, MIL neurons and other Lgr3-positive neurons (arrowheads) marked by sfGFP::Lgr3 expression (anti-GFP, green) do not express CRE-luciferase (anti-luciferase, magenta) following ectopic Dilp8 expression (*tub>dilp8b)*. CRE-luciferase staining in clock neurons (arrows) serves as internal control. Background anti-luciferase staining in glial cells is also detectable throughout the CNS. f, esophageal foramen. Midline is labeled with a dashed line. Scale bars = 50 µm.

Supplementary Figure 15

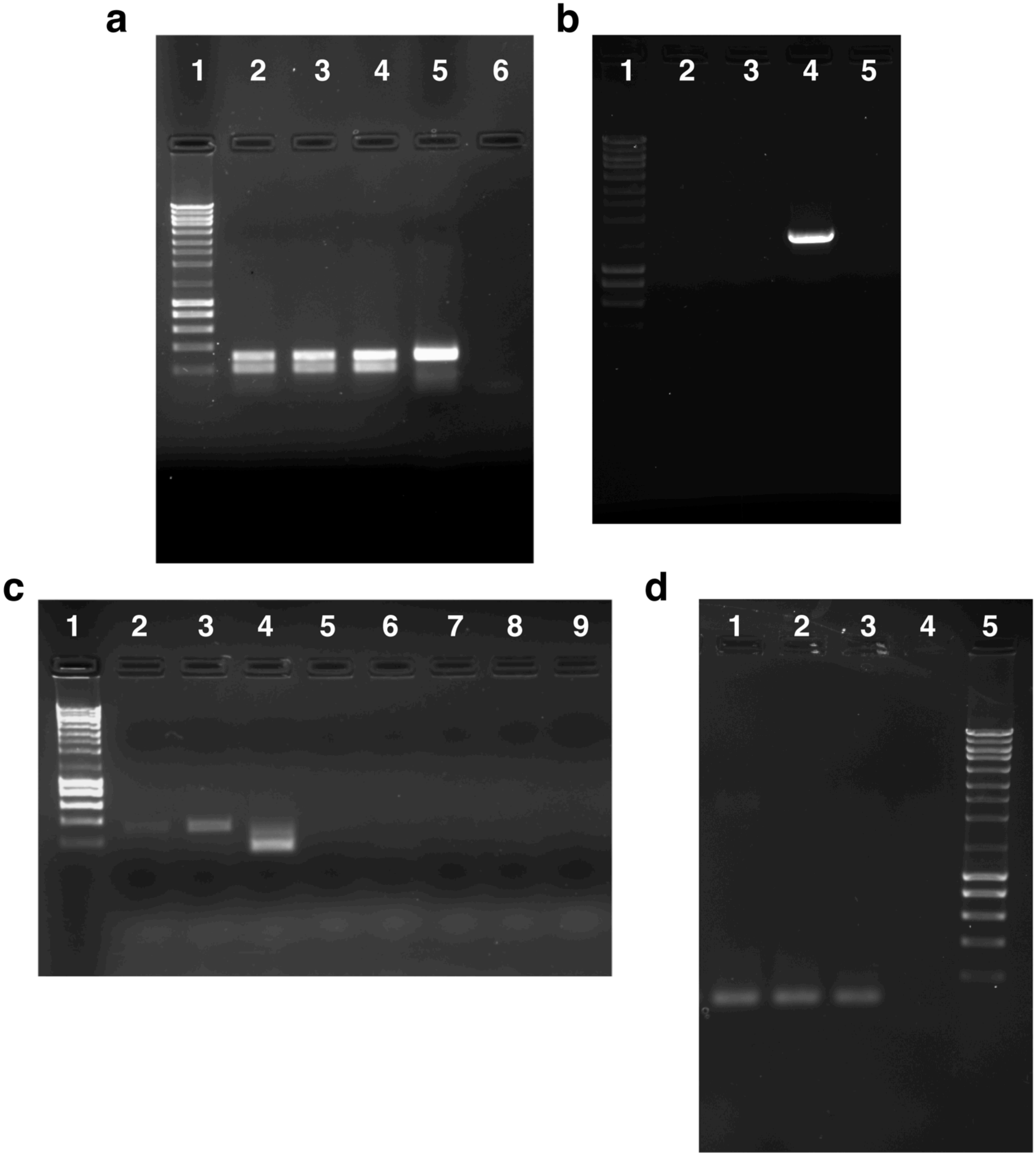

Supplementary Figure 15: Full gels for Supplementary Figures 1 and 2. a, Full agarose gel for Supplementary Fig. 1b. Lanes 1-6, markers, w^{1118}, $Lgr3^{MB06848}$, $Lgr3^{+/+}$, $Lgr3^{ag}$, dH_2O, respectively. **b**, Full agarose gel for Supplementary Fig. 1c. Lanes 1-5, markers, $Lgr3^{MB06848}$, $Lgr3^{+/+}$, $Lgr3^{ag1}$, dH_2O, respectively. **c**, Full agarose gel for Supplementary Fig. 2a and 2b. Lanes 1-5, Supplementary Fig. 2a: markers, $Lgr3^{MB06848}$, $Lgr3^{+/+}$, $Lgr3^{ag1}$, and dH_2O, respectively. Lanes 6-9, Supplementary Fig. 2b: $Lgr3^{MB06848}$, $Lgr3^{+/+}$, $Lgr3^{ag1}$, and dH_2O, respectively. **d**, Full agarose gel for Supplementary Fig. 2c. Lanes 1-5, $Lgr3^{MB06848}$, $Lgr3^{+/+}$, $Lgr3^{ag1}$, dH_2O, and markers, respectively.

Supplementary Tables

Supplementary Table 1. LRC results.

		Dilp8 vs Glycine		Dilp8 vs INS		INS vs Glycine	
**Protein**	**Accession**	**Log FC**	**adj.p**	**Log FC**	**adj.p**	**Log FC**	**adj.p**
**RYK1**	Q27324	9.179422892	0.000200131	10.05478357	0.000123552	0	NA
**SIL1**	Q9VBV5	3.46216465	0.003551489	0.180145649	0.664919456	3.219300638	0.005595375
**CTLH2**	Q9VAP3	3.36626367	0.013041077	0.819822668	0.238460354	2.477626842	0.076425902
**ITBN**	Q27591	-4.470204311	0.025904507	-0.827601414	0.664919456	-3.554393878	0.168140824
**INSR**	P09208	3.419197743	0.027455273	-0.93503451	0.225763563	4.305815168	0.023549115
**NRG**	P20241	8.384979102	0.027455273	2.177382815	0.062309474	6.034544111	0.170794493
**ITA3**	O44386	2.843122498	0.030882561	-0.013705026	0.962938305	2.807287356	0.068257205
**LACH**	Q24372	1.411934263	0.040983826	0.745425773	0.003275157	0.6327661	0.296552981
**PGAP3**	Q7K0P4	2.505140538	0.048448763	1.678665166	0.238460354	0.762280911	0.540609797
**SERR**	P18168	4.225314293	0.082118799	8.805995607	0.105875668	-4.761550987	0.296552981
**LAMB1**	P11046	1.614658346	0.08667505	0.159329106	0.631799819	1.42516401	0.168140824
**GR43A**	Q9V4K2	-3.159255375	0.091577971	-0.798140252	0.414924583	-2.296183571	0.211276594
**PERQ1**	Q7KQM6	2.293529478	0.096272321	-0.206773589	0.814859639	2.462717402	0.158569806
**GCY8E**	Q8INF0	-4.07038635	0.117585465	-0.720126905	0.667590601	-3.270346375	0.242472569
**HMH2**	P10035	-3.111886407	0.117585465	-0.38901754	0.546404538	-2.66373655	0.181282796
**ENA**	Q8T4F7	2.019338298	0.1277638	-1.399631111	0.274756118	3.400671373	0.076425902
**MAL1**	P07190	-5.548672072	0.1277638	-0.312410701	0.962938305	-5.135457221	0.347527661
**SPG20**	Q9VN45	1.44675255	0.131208357	0.739025928	0.238460354	0.673453261	0.367089194
**INTU**	Q9VPH0	-5.329717617	0.158800993	-1.310531332	0.589709295	-3.910082403	0.296552981
**CAD87**	Q9VGG5	-1.472891488	0.170162518	-0.118757156	0.962938305	-1.326940604	0.514669171
**CSUP**	Q9V3A4	-3.409233325	0.171652496	0.822217375	0.814859639	-4.181837032	0.296552981
**FAT**	P33450	-6.387174587	0.171652496	-3.065496065	0.142953906	-3.172763142	0.296552981
**PTP69**	P16620	2.296280731	0.171652496	1.939424284	0.271648086	0.293145136	0.924271636
**RL3**	O16797	-4.202156099	0.171652496	-3.558328197	0.226362165	-0.527125298	0.870217175
**FRY**	Q9VT28	-3.142075673	0.177055412	-2.251901855	0.2209807	-0.807878578	0.657530884
**PMYT1**	Q9NI63	-2.248322692	0.177055412	-0.626963188	0.615035013	-1.574433812	0.391697149
**HYD**	P51592	-2.032723528	0.185105801	-0.537362867	0.670545874	-1.453293013	0.296552981
**DPOD1**	P54358	-3.623223611	0.209528787	-1.651299449	0.241303347	-1.888514614	0.445679168
**CAD89**	Q9VEU1	-2.084439861	0.225693124	-1.300619975	0.062309474	-0.731574059	0.615953262
**ERO1L**	Q9V3A6	1.943493901	0.225693124	0.738587779	0.631799819	1.161953488	0.560695073
**FUTSC**	Q9W596	3.498966817	0.225693124	1.499431604	0.240270068	1.920143346	0.502341526
**MTRM**	Q23973	2.104029335	0.225693124	-1.37099127	0.238460354	3.454893913	0.168140824
**ORK1**	Q94526	-3.500331551	0.225693124	0.102770834	0.962938305	-3.543154344	0.158569806
**SUUR**	Q9VTE2	3.553831695	0.225693124	-0.347197419	0.619233327	3.843115646	0.284681449
**TOPI**	Q9VH70	2.20433507	0.225693124	0.636575662	0.619233327	1.52148596	0.502341526
**CBPD**	P42787	-2.874654589	0.226403404	0.271040769	0.950133194	-3.098730688	0.313768966
**SYFB**	Q9VCA5	1.785358251	0.226403404	-0.516028233	0.631799819	2.276442868	0.168140824
**GR98A**	Q9VB30	1.628552076	0.230440422	0.819657341	0.271648086	0.77046323	0.502341526
**KTU**	Q0E9G3	-1.358907984	0.230440422	-0.12335141	0.962938305	-1.210299812	0.630733411
**MYB**	P04197	-2.438699865	0.238577283	-0.341821756	0.928213919	-2.050088659	0.506354993
**NIPB**	Q7PLI2	-0.761328862	0.239492167	1.67716911	0.098722088	-2.44556613	0.125418508
**PRS4**	P48601	3.992794519	0.239492167	-0.407118648	0.780875117	4.335053331	0.296552981

**AP2A**	P91926	-2.719175205	0.255918628	-1.369755338	0.105875668	-1.285236842	0.584082162
**CRB**	P10040	3.579385189	0.277174273	-0.821858994	0.589709295	4.348651373	0.296552981
**MTH11**	P83118	-0.854186641	0.277174273	-0.113279878	0.779540879	-0.724596487	0.394570367
**NCD**	P20480	3.234827046	0.277174273	-0.015383143	0.962938305	3.193842187	0.336294069
**UGGG**	Q09332	-0.466475856	0.277174273	0.143043337	0.589709295	-0.603101792	0.296552981
**SBNO**	A8JUV0	-1.573930011	0.285804504	-1.486138523	0.482013222	-0.042216816	0.983151057
**NINAC**	P10676	1.69409701	0.351642954	4.69744471	0.006919872	-3.090042493	0.211276594
**FITM**	Q9VRJ2	1.364139257	0.37892656	0.401611458	0.589709295	0.933798558	0.583361931
**SNP25**	P36975	-1.39383831	0.37892656	-0.923120309	0.007333585	-0.435132893	0.809796864
**ATPB2**	Q24048	0.933126307	0.392098805	-0.488441391	0.780875117	1.411188571	0.296552981
**CSTN1**	Q9V498	3.048450829	0.392098805	0.786950729	0.635733105	2.198641718	0.584082162
**CUE**	Q95RU0	-1.620044462	0.392098805	0.801886803	0.271648086	-2.403351198	0.296552981
**HR4**	Q9W539	-1.389947368	0.392098805	4.092561478	0.163915894	-5.507935196	0.168140824
**ITBX**	P11584	0.539439121	0.392098805	0.193121545	0.241303347	0.334539951	0.610033921
**KCNAE**	Q02280	4.497186973	0.392098805	1.091494034	0.631799819	3.313805732	0.561093726
**MYSA**	P05661	1.483773938	0.392098805	1.148496354	0.062309474	0.295381671	0.913657549
**SCN60**	Q9W0Y8	-0.963233127	0.392098805	2.800381566	0.098722088	-3.780800613	0.158569806
**TENM**	O61307	1.179217992	0.392098805	0.911914341	0.431238807	0.235606938	0.916408976
**HIG**	Q09101	-1.715697832	0.41665648	-1.897040414	0.225763563	0.234388496	0.931484021
**SEM2A**	Q24323	-0.980859576	0.434198047	0.364150103	0.269030188	-1.332285813	0.383066739
**GPI8**	Q8T4E1	0.606083733	0.436942331	1.806925613	0.117477228	-1.233393313	0.296552981
**FSH**	P13709	-1.141661226	0.521444508	0.244303095	0.679762713	-1.368972928	0.506354993
**IDGF1**	Q8MM24	0.971732334	0.546122252	2.124002798	0.296564694	-1.195067252	0.623222612
**OCTL**	Q95R48	0.889062409	0.546122252	0.454142237	0.369469849	0.413858484	0.812336862
**FOSLA**	P21525	-0.59581789	0.551130531	2.169667044	0.416526124	-2.781430878	0.336294069
**OSA**	Q8IN94	1.260901556	0.551130531	-0.061105065	0.962938305	1.300704572	0.610033921
**FOXF**	Q9VS05	0.766228852	0.634996972	-2.613369785	0.062309474	3.397956508	0.168140824
**LAMA**	Q00174	0.733345257	0.634996972	1.323505663	0.431238807	-0.619062939	0.809796864
**CONT**	Q9VN14	0.101091371	0.648623559	0.79652434	0.232442492	-0.706878594	0.296552981
**HSP83**	P02828	-0.644616016	0.676713235	-0.453787445	0.667590601	-0.174044883	0.931484021
**A36DE**	Q9V3R1	0.906902324	0.69447483	-1.386264425	0.062309474	2.294155167	0.506354993
**ATPB1**	Q24046	1.705308152	0.737759906	-0.279314966	0.962938305	1.958202825	0.506354993
**CADN**	O15943	0.79623769	0.770088949	-1.606007689	0.238460354	2.40783857	0.41418098
**EXOC4**	Q9VNH6	-1.259666184	0.772528328	2.24340327	0.837859622	-3.508305186	0.472804709
**INHB**	O61643	0.137418356	0.772528328	-0.403296211	0.681557322	0.543212346	0.598052865
**MICAL**	Q86BA1	-0.311397579	0.772528328	-0.354559902	0.589709295	0.052914636	0.983151057
**SC5A7**	Q9VE46	1.129178295	0.772528328	0.439064083	0.270053521	0.665037753	0.913657549
**VEIN**	Q94918	-0.331835854	0.772528328	-0.314055129	0.385410189	-0.008163243	0.992245371
**FAS1**	P10674	0.353261771	0.811803448	0.179369942	0.849850517	0.165536257	0.952194544
**PDM1**	P31368	-0.7006991	0.811803448	-0.097747241	0.962938305	-0.589513758	0.913657549
**GMDS**	Q9VMW9	-0.376108744	0.842603652	-0.412784147	0.837859622	0.048266528	0.983151057
**LAMC1**	P15215	-0.296259394	0.869262915	-0.232231406	0.679762713	-0.056026699	0.983151057
**CRQ**	Q27367	-0.175143272	0.898065522	-3.802468926	0.232442492	3.676589902	0.170794493
**OB56A**	Q9V8Y2	0.134739038	0.898065522	-0.614169803	0.631799819	0.754015698	0.642167793
**SUR**	Q9VL32	0.047217413	0.966223798	-1.590113526	0.098722088	1.655826229	0.362806143

LRC results for the Log_2 Fold Change and FDR-adjusted *p* value for the enrichment of each detected protein for the experiments: Dilp8 vs Glycine, Dilp8 vs insulin (INS) and INS vs Glycine controls. Protein names are given in the first column. Data are ordered based on the Dilp8 vs Glycine experiment.

Supplementary Table 2. Raw data for LRC assays.

				Dilp8			Glycine			INS		
**Protein**	**Sequence**	**z**	**m/z**	**REP1**	**REP2**	**REP3**	**REP1**	**REP2**	**REP3**	**REP1**	**REP2**	**REP3**
**A36DE**	GSNPSNLFII	2	0.8627	0.4084	0.3897	0.4383	0.0383	0.0843	1.1580	1.1593	0.0000	1.1053
**AP2A**	LNETLETILNK	2	1.0469	0.4726	0.2889	0.2644	1.0441	10.6511	0.4117	1.1254	0.4164	1.0852
**ATPB1**	DYNHTEGR	2	0.8058	3.3220	2.5620	0.0031	0.0846	0.0856	0.0393	1.6215	0.6128	0.0401
**ATPB2**	IYGWIPEYYNR	2	1.1970	0.7275	0.7024	2.2970	0.4088	0.3923	0.4235	0.7033	2.2636	1.4828
**CAD87**	YTLIGENNK	2	0.8553	0.1043	0.0418	0.0689	0.1320	0.1764	0.0987	0.1580	0.0126	0.1990
**CAD89**	MLNLTVTDIDGLK	2	1.1767	0.7201	0.2387	0.3174	1.1102	0.3977	1.4089	1.0141	0.5185	1.0978
**CAD89**	NSSQSLVMSR	2	0.9138	0.2150	0.1971	0.1719	2.1436	2.3788	0.1194	0.4574	0.4195	0.5524
**CADN**	QFAINQNGSVT	2	0.9584	0.7803	0.3333	0.9740	0.9023	0.0231	0.8284	2.5428	0.7593	2.8088
**CBPD**	LDGANHSTY	2	0.7944	0.0811	0.0250	0.1038	0.0791	0.3660	1.0141	0.1127	0.0082	0.1383
**CONT**	QPNDTTFDVNK	2	1.0249	0.7665	0.8292	0.5971	0.0514	0.0423	0.1080	0.2934	0.2514	0.3290
**CONT**	LTVHNTSMR	2	0.8732	0.2109	0.2136	0.2141	0.8438	0.6539	2.5800	0.2281	0.0629	0.2852
**CRB**	NGSTCQNGFNASTGN	2	1.2431	1.4786	1.0115	3.7129	0.4482	0.0061	0.4162	3.2686	1.2921	5.0763
**CRQ**	PCGIVNGTTGDMFPPK	2	1.3860	0.0915	0.7566	0.0668	0.2733	0.0720	0.1204	1.1193	6.6394	1.3001
**CSTN1**	DGFTHIN	2	0.6532	0.2106	0.3706	0.5544	0.2910	0.0009	0.0884	0.1220	0.4255	0.1379
**CSUP**	ANENFDPQK	2	0.8634	0.1276	0.0286	0.0929	1.1587	0.1183	1.0397	0.5078	0.0086	0.0148
**CTLH2**	FYNGTNDAQLLGEK	2	1.2749	2.2520	1.5480	1.2830	0.0991	0.1478	0.1106	0.9930	0.9382	0.6433
**CUE**	VANLSEEAR	2	0.8034	0.2649	0.4134	0.4501	0.6211	4.0953	0.2411	0.1772	0.1990	0.2267
**DPOD1**	FNGTSNGHAK	2	0.8391	0.1244	0.0413	0.0234	0.4632	2.0796	0.0923	0.2215	0.0695	0.2396
**ENA**	KCVSPSNPSVN	2	0.9665	1.0081	0.5043	1.3506	0.2601	0.1308	0.1182	3.4048	0.8058	3.4000
**ERO1L**	SIYLENCFGGNNETANK	2	1.5678	0.9568	0.6387	0.5588	0.3525	0.1489	0.0452	0.1978	0.8513	0.3467
**EXOC4**	LPNWTDLK	2	0.8018	0.3610	0.5802	0.0012	0.2064	0.0180	0.2933	0.0000	0.0000	0.0146
**FAS1**	FSHFNDQLNNTQR	2	1.3162	0.5484	0.4411	0.5006	0.2152	0.0092	0.0237	0.1371	0.3832	0.3521
**FAS1**	FSHFNDQLNNTQR	3	0.8780	1.2155	1.1374	1.0016	1.1239	3.0073	0.2012	0.7503	1.4848	0.8946
**FAT**	YNLTVVAMDQGTPARTTTAH	2	1.7561	0.0835	0.0000	0.0748	8.3392	24.9688	0.6466	1.2487	0.2330	0.9350
**FITM**	SGSNMNFRPGGPDITR	2	1.3982	1.5341	1.3436	1.0463	0.2176	1.2899	0.1924	1.0130	1.0505	0.6530
**FOSLA**	NFSNVLAAVSSSR	2	1.0980	0.1527	0.2146	0.2080	0.1000	0.2183	0.3926	0.0952	0.0052	0.1513
**FOXF**	SLNGSESSPPSQNH	2	1.1701	3.0017	1.0830	2.5392	3.7318	0.5092	0.3797	11.3310	8.3208	12.8823
**FRY**	GGNPSGGSPGQR	2	0.8699	0.1712	0.0626	0.1072	0.1625	0.0872	0.1177	0.2442	0.1178	0.2845
**FRY**	RISTLNASGYQNVTIFSYN	2	1.7447	0.1166	0.1034	0.8946	26.8471	0.3712	46.6296	0.6769	4.9898	3.7288
**FSH**	GGAGAAGGGNASK	2	0.7920	0.3120	0.2344	0.3035	0.2152	2.1299	0.2138	0.1894	0.2570	0.2379
**FUTSC**	LNNSNVQGLGAVVSRK	3	0.8976	2.4644	4.5504	2.4220	0.5444	0.5896	0.0259	0.8113	1.5944	0.6515
**GCY8E**	SNSTGHVFMR	2	0.9227	0.1970	0.0763	0.1917	0.6241	5.3270	1.6836	0.3831	0.0601	0.5178
**GMDS**	EVGVENGTGIVR	2	0.9990	1.4040	0.5264	0.1357	1.3086	0.0816	0.7673	0.4795	1.0843	0.3640
**GPI8**	DNTSVLPEGFVDAAQR	2	1.3958	0.4986	0.3472	0.2449	0.0985	0.0632	0.0500	0.0787	0.0210	0.1502
**GPI8**	INVTLANE	2	0.7101	0.8447	0.6179	0.1475	0.4467	0.2142	1.2218	0.4891	0.0838	0.1433
**GR43A**	NVSVNRPA	2	0.6963	0.2281	0.1200	0.1191	1.8884	0.3275	1.4080	0.4008	0.1058	0.3677
**GR98A**	FSAGGMVDIN	2	0.8342	0.1355	0.3199	0.1288	0.0625	0.0135	0.0742	0.1165	0.0768	0.1055
**HIG**	NISVTPIPPN	2	0.8546	0.2506	0.0570	0.1372	1.7234	0.2127	0.0725	0.7866	0.2393	0.4782

**HMH2**	LNLTDAQV	2	0.7101	0.3932	0.3372	0.3413	1.2284	6.9789	1.4684	0.5995	0.2271	0.6252
**HR4**	SSSSSSGNGSGGK	2	0.8926	0.1908	0.3616	0.1697	1.5019	0.0950	0.5386	0.0402	0.0021	0.0295
**HSP83**	ELISNASDALDK	2	1.0363	0.9598	0.9056	0.5902	0.4738	0.0986	1.4152	0.4145	0.7443	0.4466
**HSP83**	SNASDALDKIR	2	0.9665	0.2176	0.0421	0.2262	1.7286	0.2038	0.0947	0.4118	0.1688	0.5162
**HYD**	ADSNQSTTR	2	0.7961	0.1602	0.0957	0.2884	0.3106	0.3803	0.9532	0.3160	0.0954	0.4070
**IDGF1**	LSQNASAQYR	2	0.9243	0.2897	0.2376	0.0001	0.0257	0.1467	0.0072	0.1336	0.0078	0.0140
**IDGF1**	LSQNASAQYR	2	0.9243	0.0082	0.0161	0.1934	0.0518	0.1720	0.0231	0.0081	0.0021	0.1091
**INHB**	KVNGINGTQM	2	0.8635	0.5867	0.5412	0.6962	0.3897	0.5313	0.3219	1.0502	0.2781	1.3949
**INSR**	VEIGEPQKPSNATIVFK	3	1.0058	0.4074	0.5328	0.2281	0.0431	0.0204	0.0162	0.6490	0.6723	0.6406
**INTU**	EPGNSSRL	2	0.6987	0.0614	0.0147	0.0147	0.3323	5.8603	0.1754	0.1283	0.0116	0.1494
**ITA3**	VAGSTAIIPIINVTSLK	2	1.3781	0.6962	0.7001	0.4384	0.0149	0.0025	0.0471	0.5931	0.7129	0.5834
**ITA3**	TINETGAIYR	2	0.9243	1.1935	1.3147	1.2861	1.3497	0.2428	0.7146	0.7320	1.4257	1.0273
**ITBN**	NNVTLGK	2	0.6062	0.0678	0.0266	0.0478	0.4910	0.6808	1.0237	0.1720	0.0169	0.1729
**ITBX**	FANDTSCR	2	0.7887	0.6693	0.9862	0.5973	0.1307	0.0173	0.3021	0.3860	0.6125	0.4816
**ITBX**	NCTQFVPVGVEK	2	1.1191	1.2210	1.2130	1.3795	0.6280	1.0365	0.6720	0.7270	1.1027	0.9181
**ITBX**	LDNDSSNVVELVK	2	1.1629	2.5761	2.0784	1.8569	8.9086	1.2295	5.3919	1.9554	2.7163	2.1686
**KCNAE**	GLVAPQN	2	0.5681	1.5261	3.7981	5.1304	1.2675	0.0008	0.8337	0.4955	3.4066	1.2408
**KTU**	GSALKQEENPSR	2	1.0688	0.1690	0.1144	0.0986	0.1679	0.4966	0.1462	0.3951	0.0147	0.4118
**LACH**	RPPVISDNSTQSVV	2	1.2173	1.7214	1.4450	1.2577	0.2975	0.3077	0.0839	0.6190	0.6517	0.6222
**LACH**	RPPVISDNSTQSVVASE	2	1.4502	1.3480	1.3667	1.1154	0.3058	0.6877	0.4914	0.9131	1.2997	0.9614
**LACH**	RPPVISDNSTQSVVA	2	1.2749	0.6951	0.7091	0.6793	0.1007	1.1232	0.2503	0.3636	0.2624	0.3406
**LAMA**	ISAVNNATEHQLK	2	1.1573	0.1336	0.1306	0.2172	0.0462	0.2139	0.0301	0.0741	0.0792	0.1049
**LAMA**	QVNQTLANAF	2	0.8984	0.0990	0.0695	0.0662	0.0833	0.0061	0.0636	0.1804	0.0033	0.0198
**LAMB1**	VNNLQSIANATK	2	1.0339	0.4199	0.4757	0.4222	0.0004	0.0827	0.0019	0.1590	0.3305	0.1588
**LAMB1**	GEANNLQSATSATNQTLTDR	2	1.6985	0.4503	0.3959	0.3270	0.0239	0.0182	0.0971	0.4007	0.3277	0.2726
**LAMB1**	YLNQTNANIK	2	0.9576	0.2962	0.3562	0.4103	0.0345	0.7691	0.3468	0.2576	0.2537	0.3977
**LAMB1**	LNASEAYEK	2	0.8326	0.3361	0.3556	0.3706	0.5333	0.3508	0.3192	0.2501	0.2594	0.2880
**LAMB1**	NIEGALNLTR	2	0.8951	0.5385	0.1918	0.6732	0.8883	1.2246	0.1543	0.8912	0.3755	1.0648
**LAMC1**	LFNLSQTLDEIAR	2	1.2344	0.3284	0.3087	0.2128	0.7117	0.0305	0.6433	0.4070	0.3277	0.2236
**MAL1**	NGSHIPF	2	0.6273	0.0381	0.0047	0.0028	0.1954	1.1542	0.0810	0.0678	0.0002	0.0808
**MICAL**	STGPNSSTTGNVSK	2	1.0858	0.9035	1.0193	0.7802	1.1623	1.6597	2.5654	0.9749	1.3647	0.9057
**MICAL**	LLNPSSDI	2	0.6979	0.6783	0.3496	0.7234	0.2046	0.0187	1.5410	1.2189	0.4787	1.2485
**MICAL**	TTSNLSSLTR	2	0.8773	0.2049	0.6860	0.1562	0.2547	0.9770	0.1489	0.2279	0.2283	0.2751
**MTH11**	QLNGSVIK	2	0.6980	0.9030	0.8776	0.9531	0.8360	2.0714	1.0956	1.0393	0.5819	1.2076
**MTRM**	MENSRTPTNKTK	2	1.1767	0.6007	0.3439	0.5932	0.0290	0.0961	0.2025	1.5636	0.4900	2.1737
**MYB**	SVNASGSDLK	2	0.7945	0.2197	0.0857	0.2047	2.2619	0.1277	0.7782	0.4955	0.0250	0.5950
**MYSA**	NKDPLNDTVVDQFK	2	1.3260	1.2694	1.7835	1.2080	0.5212	0.0729	1.2429	0.5851	0.5285	0.6163
**NCD**	NSSTQSNNSGSFDK	2	1.1977	0.4538	0.5865	0.3201	0.0277	0.3020	0.0049	0.6815	0.2537	0.4207
**NINAC**	INMNMSFPR	2	0.9153	0.3181	0.4959	0.3257	0.0638	0.0233	0.3491	0.0107	0.0136	0.0200
**NIPB**	QNHSSNQHIK	2	0.9697	0.2779	0.3087	0.2848	0.6036	0.3414	0.2238	0.1219	0.0432	0.1310
**NRG**	GDTGNYGCNATNSLGY	2	1.3510	1.0095	1.0877	0.8017	0.0000	0.0033	0.0011	0.3075	0.0000	0.1628
**OB56A**	SSLNLSDEQK	2	0.9105	0.2334	0.1054	0.1534	0.1670	0.1922	0.0335	0.4170	0.0780	0.3809

**OCTL**	CSYYDVDYTEEYLNGSIPR	2	1.8891	3.3384	3.6679	2.7010	1.2619	0.0579	1.7711	3.1430	2.7354	2.6890
**OCTL**	NLSYPENER	2	0.9113	0.1495	0.1131	0.2730	0.0651	0.2455	0.2596	0.0699	0.0976	0.0916
**ORK1**	NTTTQDEIL	2	0.8407	0.0133	0.0044	0.0878	0.2403	0.0511	0.1983	0.0159	0.0073	0.0424
**OSA**	GMPNHTG	2	0.5802	0.6565	0.7451	0.1918	0.0680	0.8053	0.0505	0.5112	0.9425	0.1781
**PDM1**	NGGALNLTSDNSR	2	1.0712	0.0185	0.0760	0.0147	0.0328	0.3273	0.0030	0.0157	0.1024	0.0168
**PERQ1**	GALDWNGTPSSSPR	2	1.1734	10.6775	8.0681	12.8211	1.6567	0.8870	2.8570	9.4595	12.3311	8.5088
**PGAP3**	TNCSADGLEIQEQAVK	2	1.4315	0.2000	0.1218	0.1915	0.0328	0.0196	0.0136	0.0256	0.0699	0.0766
**PMYT1**	SLMNFSML	2	0.7790	0.0714	0.0549	0.0503	0.5833	0.1807	0.0729	0.1441	0.0315	0.1620
**PRS4**	MGQNQSA	2	0.5980	6.4267	8.7001	2.4304	1.0211	0.0135	0.9223	8.2162	8.6371	2.7742
**PTP69**	DFINGSHTSY	2	0.9267	1.2377	1.1661	0.9207	0.0285	0.0539	0.0481	0.3574	0.6930	0.3523
**PTP69**	QEVHSDNVTR	2	0.9486	0.3005	0.3851	0.0094	0.0008	0.1472	0.0176	0.1309	0.0125	0.0185
**PTP69**	QEVHSDNVTR	2	0.9486	0.0007	0.0006	0.2288	0.0278	1.9128	0.0744	0.0000	0.0028	0.1577
**RL3**	NNASTEYDLTDK	2	1.1133	0.0318	0.0040	0.0228	0.0438	0.4924	0.2871	0.3513	0.0375	0.3819
**RYK1**	EVLPRPILNISR	2	1.1427	1.0604	1.6364	0.8641	0.0000	0.0000	0.0000	0.0000	0.0000	0.0000
**SBNO**	GNNSMMEAVQKL	2	1.0874	0.1889	1.1936	0.1628	0.8844	0.6759	0.6374	0.5574	1.6181	0.7034
**SC5A7**	KMLDNATGVKP	3	0.6449	0.3127	0.3380	0.2162	0.1523	1.6263	0.0038	0.1830	0.1827	0.2398
**SCN60**	KTAMNNT	2	0.6468	0.7091	0.8523	0.7743	1.0442	2.7616	0.5200	0.1700	0.0361	0.1971
**SEM2A**	TDLYNTSAK	2	0.8229	0.1576	0.1475	0.1707	0.6567	0.3961	0.7846	0.1488	0.1083	0.1361
**SEM2A**	VNLQNISSSNCNR	2	1.2229	0.2549	0.2832	0.2192	0.0177	0.6239	0.2343	0.2166	0.1930	0.1230
**SERR**	NLSGVCK	2	0.6322	0.4926	0.6252	0.5551	0.0085	0.0547	0.0205	0.0003	0.0216	0.0003
**SIL1**	DVLLPIVVNDTSTSLR	2	1.4146	4.1205	2.5906	2.8215	0.2105	0.1796	0.2431	3.4787	1.6330	2.4793
**SNP25**	KNLSGMEK	2	0.7499	0.0891	0.0724	0.0723	0.0775	0.0679	0.5403	0.1628	0.1154	0.1616
**SPG20**	RPPLLAENPSTQ	2	1.0745	13.9661	15.3936	18.5497	4.7011	7.1307	2.9193	8.3304	7.8379	7.6220
**SUR**	WLLNDTIR	2	0.8375	0.6292	0.3902	0.7372	0.3346	0.1263	1.4416	2.2505	0.9800	1.7064
**SUUR**	LNSSGEVSPVQP	2	0.9860	6.0618	7.6581	5.7109	0.9852	0.0394	1.6492	6.8029	8.2066	5.9694
**SYFB**	EQGDVAAAANASEEII	2	1.2749	2.6241	2.3919	5.8931	0.7641	0.3151	1.5471	3.2935	3.8176	5.6179
**TENM**	LETFNESR	2	0.8091	1.0124	1.0203	1.1320	0.0101	0.1051	0.0847	0.5092	0.4310	0.4861
**TENM**	LGGMFNISTHE	2	0.9795	0.0439	0.0355	0.0117	0.1346	0.0396	0.0413	0.0342	0.0035	0.0342
**TOPI**	NNPTKPI	2	0.6371	0.7425	0.5503	0.5298	0.2344	0.0000	0.0446	0.3280	0.6565	0.2155
**UGGG**	SFDHIFPGSENNTR	3	0.8780	2.4209	2.7286	2.0711	0.1766	0.2077	0.0677	1.8851	2.5167	2.1154
**UGGG**	SFDHIFPGSENNTR	2	1.3162	1.4169	1.4759	2.0041	0.6698	0.1078	1.2432	1.3810	1.2578	1.4561
**UGGG**	HIFPGSENNTR	2	1.0330	0.6851	0.5742	0.8195	9.6049	1.7707	4.3622	0.5154	0.4245	0.6279
**VEIN**	LQFALSN	2	0.6444	0.1753	0.1295	0.1522	0.2125	0.0387	0.2882	0.2178	0.1225	0.2276

Identified peptide sequence and raw peptide counts for each repeat (REP1-3) for each condition assayed (Dilp8, Glycine and INS). z, charge. m/z, mass-to-charge ratio.
